# Abstracts from the 12th International Conference for Healthcare and Medical Students (ICHAMS)

**DOI:** 10.1186/s12919-023-00277-8

**Published:** 2023-10-26

## 2022 ICHAMS Presentations

### PP1: Vitamin-D receptor gene polymorphisms (ApaI and TaqI) and circulating Vitamin D 25-Hydroxy Metabolites in Patients with Cardiovascular Diseases

#### Mohamed Abouzid^1^ , Marlena Kruszyna^2^ , Paweł Burchardt^2^ , Łukasz Kruszyna^3^ , Franciszek K. Główka^1^ , Marta Karaźniewicz-Łada^1^

##### ^1^Department of Physical Pharmacy and Pharmacokinetics, Poznan University of Medical Sciences, 6 Święcickiego Street, 60-781 Poznan, Poland; ^2^Department of Hypertension, Angiology, and Internal Medicine, Poznan University of Medical Sciences, Długa ½, 60-848 Poznan, Poland; ^3^Department of Vascular and Endovascular Surgery, Angiology and Phlebology, Poznan University of Medical Sciences, Długa ½, 60-848 Poznan, Poland

###### **Correspondence:** Mohamed Abouzid

*BMC Proceedings 2023*, **17(Suppl 17):**PP1

**Introduction**: The relationship between vitamin D receptor (*VDR*) polymorphisms and cardiovascular disease (CVD) is unclear. This study explores the correlation between *VDR* genotypes, plasma concentrations of vitamin D metabolites, and CVD occurrence and metabolic disorders.

**Method**: Fifty-eight patients with CVD were included. *ApaI* rs7975232 and *TaqI* rs731236 were genotyped using PCR-RFLP method. Circulating 25-hydroxyvitamin-D2, 25-hydroxyvitamin-D3, and 3-epi-25-hydroxyvitamin D3 were measured by a validated UPLC-MS/MS method.

**Results**: *ApaI* polymorphism frequencies were significantly (*p* = 0.01) associated with hypertension cases (GG 71%, GT 48%, TT 93%). Patients with *ApaI*-GG genotype had significantly (*p* < 0.01) higher plasma levels of 25(OH)D3 and 3-epi-25(OH)D3 compared with *ApaI*-GT carriers. Backward stepwise regression shows that hypertension can be predicted by BMI (OR=2.11, 95%CI=0.08–1.41, *p*=0.03), HbA1C[%] (OR=32.39, 95%CI= 0.17–6.79, *p*= 0.04), while *ApaI*-GT was protective (OR=0.05, 95%CI=−5.92-(−0.02), *p*= 0.03). For *TaqI* genotypes, significant differences between the values were found in BMI (*p* = 0.04), and obesity incidence (CC 22%, TC 50%, TT 12%; *p*<0.01) and hypercholesterolemia (CC 67%, TC 45%, TT 69%; *p*=0.05). 3-epi-25(OH)D3 levels were significantly lower in *TaqI*-TC genotype compared with *TaqI*-CC genotype (*p*=0.03).

**Discussion**: *ApaI*-GT genotype can be protective against hypertension. *Taq*-TC and *Taq*-TT genotypes were associated with a higher risk of obesity and hypercholesterolemia, respectively. *ApaI*-GT and *Taq*-CC genotype were associated with higher 25-hydroxyvitamin-D levels. Further research is required to confirm these outcomes and to investigate their potential mechanisms.

**Acknowledgements**

Mr. Mohamed Abouzid is a participant of STER Internationalization of Doctoral Schools Programme from NAWA Polish National Agency for Academic Exchange No. PPI/STE/2020/1/00014/DEC/02.

### PP2: Pulmonary embolism risk stratification in cardiovascular patients and its preventive measures

#### Hossam Bajbouj, Anna Tytova

##### Kharkiv National Medical University

###### **Correspondence:** Hossam Bajbouj; Anna Tytova

*BMC Proceedings 2023*, **17(Suppl 17):**PP2

**Introduction:** The prevalence of pulmonary embolism is estimated to be 60-70 per 100,000, Nevertheless, actual numbers are exponentially higher due to silent PE in 40%-50% of cases. The study focused on the association between cardiovascular disorders and PE.

**Methods:** The study population consisted of 58 patients with cardiovascular pathology (37 female and 21 male). To estimate the risk of pulmonary embolism in cardiovascular patients; PE clinical score was used: Geneva scale and Wells scale.

**Results:** According to the risk of developing PE, patients were divided into 3 groups. The 1^st^ group –low risk– 12 patients (5 men and 7 women,56,8±3,4 years of age), among them 42% with atherosclerotic cardiosclerosis, 25% with stable angina and 33% with NQMI, the 2^nd^ group -intermediate risk– 28 patients (10 men and 18 women, 62,5±4,6 yrs.): 53% had stable angina, 7% unstable angina, 10% QMI, 20% QMI and 10% atherosclerotic cardiosclerosis; 3^rd^ group –high risk- 9 patients ( 4 men and 5 women, 64,6±6,7 yrs.) 14% stable angina, 14% unstable angina, 14% QMI and 58% atherosclerotic cardiosclerosis. It was found that complications such as atrial fibrillation, extrasystolic arrhythmia, COPD, chronic bronchitis, and autoimmune thyroiditis increase the risk of PE in patients with cardiovascular diseases. The high and intermediate-risk groups were mostly seen in patients with NQMI and QMI. However, the highest number of cases with intermediate-risk were among patients with stable angina and atherosclerotic cardiosclerosis; also, atherosclerotic cardiosclerosis patients showed a 58% of high risk of PE. This confirms propitious anticoagulant therapy in patients with unstable angina, NQMI, and QMI, but insufficient anticoagulation in patients with stable angina and atherosclerotic cardiosclerosis.

**Discussion:** Based on the above-mentioned data, more intensive anticoagulant/antiplatelet therapy should be introduced into treatment protocols of patients with stable angina and atherosclerotic cardiosclerosis due to their higher risk of developing PE.

### PP3: An Investigation into the Transmissibility of Covid-19 from Vaccinated, Unvaccinated and Partially Vaccinated cases in South West Ireland 2021

#### Eoghan Mooney^1^, Michael Hanrahan^2^, Elena Khoo^1^, Finbarr Condon-English^1^

##### ^1^University College Cork; ^2^Department of Public Health, HSE-South, Cork, Ireland

###### **Correspondence:** Eoghan Mooney

*BMC Proceedings 2023*, **17(Suppl 17):**PP3

**Background**: As the COVID-19 pandemic continues globally, key questions pertaining to the transmissibility of the virus remain unanswered. One major consideration to future health policy worldwide is an understanding of the extent that vaccines might reduce the likelihood of transmission from infected cases who are fully vaccinated compared with unvaccinated or partially vaccinated cases. An understanding of how transmissibility may be influenced by the severity of symptoms in infected cases may also inform pragmatic approaches to risk reduction in the future.

**Methods**: A retrospective cohort study was undertaken to ascertain the transmissibility of Covid-19 from randomized adult samples from the regions of Cork and Kerry to compare the effective R numbers of index cases between groups and relative risk of close contacts contracting the virus from randomized groups of vaccinated (n=74), partially vaccinated (n=100), and unvaccinated (n=100). Data was extracted from the National Case Tracker Customer Relationship Management (CRM) system.

**Results**: Fully vaccinated cases were associated with 56% fewer secondary cases compared to unvaccinated adults (95% CI 0.28 – 0.70). Being a close contact of a fully vaccinated positive case carries a 42% lower risk of contracting COVID-19 compared to being a close contact of an unvaccinated case (95% CI 0.35 – 0.97). There were no differences observed between unvaccinated and partially vaccinated cases.

**Conclusion**: The findings of this study enhance the understanding of the effect vaccination may have on transmission of COVID-19 by indicating the benefits that immunisation appears to confer on the vaccinated compared to the unvaccinated.

### PP4: Urinary tract infection caused by an emerging multi drug resistant Myroides species- A case report

#### Ellora Pandey, Shahzad Mirza

##### Dr. D.Y. Patil Medical College, Hospital and Research Centre, Dr. D. Y. Patil Vidyapeeth

###### **Correspondence:** Ellora Pandey

*BMC Proceedings 2023*, **17(Suppl 17):**PP4

**Introduction:** The genus Myroides comprises Gram-negative, non-motile, and non-fermenting bacteria. Though not often considered pathogenic, Myroides species have rarely been associated with Hospital Associated Infections (HAI) in immuno-compromised, diabetics, and prolongedly catheterized patients. The aim of the report is to present a very rare cluster of Urinary Tract Infections (UTIs) caused by a multidrug resistant Myroides spp. which were difficult to treat.

**Methods:** Aseptically collected mid-stream urine specimens from inpatients admitted in the Intensive Care Units were processed as per standard laboratory protocols. Identification and Antibiotic Susceptibility Testing (ABST) was performed on VITEK® 2C (BioMérieux, France), as per Clinical and Laboratory Standards Institute (CLSI).

**Results:** We report three separate cases of UTI in patients with prolonged catheterization as well as underlying co-morbidities such as diabetes mellitus. Extensive drug resistance was seen in all three cases on urinary culture. ABST revealed sensitivity to only Minocycline, the bacteria being resistant to beta-lactams, beta-lactamase inhibitors, monobactams, aminoglycosides, fluoroquinolones, carbapenems, polymyxins, and sulfonamides. All the patients failed the initial empirical therapy, as this organism was extensively drug resistant, and had to undergo prolonged treatment and hospital stay. Two patients were successfully treated with Minocycline which was confirmed by a negative urine culture, while one patient did not have a favourable outcome.

**Discussion:** Though uncommon, clinicians should be cognizant of the ability of Myroides to cause prolonged UTI outbreaks, especially in the immuno-compromised patients. Selection of appropriate antibiotic therapy to treat this infection is difficult due to the organism’s intrinsic resistance to many antibiotic classes and the production of biofilm. Accurate identification of Myroides spp. is necessary for rapid, prompt, and definitive treatment considering their extensive resistance.

### PP5: Effects of LCS-1269 on epigenetic regulation of transcription

#### Valeriia Popova^1^, Varvara Maksimova^1^, Olga Usalka^1^, Guzel Khayrieva^2^, Gusel Sagitova^2^, Roman Zenkov^1^, Vera Eremina^1^ , Lidia Ektova^1^ , Marianna Yakubovskaya^1^ , Kirill Kirsanov^1^

##### ^1^ N.N. Blokhin National Medical Research Center of Oncology (N.N. Blokhin NMRCO), Mendeleev University of Chemical Technology; ^2^ I.M. Sechenov First Moscow State medical University (Sechenov University)

###### **Correspondence:** Valeriia Popova

*BMC Proceedings 2023*, **17(Suppl 17):**PP5

**Introduction:** Previously we showed, that novel indolocarbazole derivative LCS-1269 synthesized at the N.N.Blokhin NMRCO has a strong antitumor effect against neoplasms of various nosology *in vivo* and *in vitro*. One of the important details of the antitumor activity of anticancer drugs is their effect on the epigenetic regulation of transcription.

**The aim** of this research was to study the effects of LCS-1269 on the main mechanisms of epigenetic regulation of transcription.

**Materials and methods:** The analysis of integral epigenetic activity was carried out using the HeLa TI test system using FACS. Effects of LCS-1269 on histone modifications and HDAC1 protein were determined with Western blotting. Changes in the amount of mRNA of enzymes HDAC1, HDAC3, DNMT1, DNMT3A, and DNMT3B were analyzed by real-time RT-PCR. The level of integral DNA methylation was studied using methyl-sensitive restriction analysis (MspI/HpaII) and ELISA sensitive to cytosine methylation.

**Results:** It was shown that LCS-1269 causes increase the number of GFP-positive cells in the HeLa TI test system by more than 3 times. Further it was demonstrated that LCS-1269 increased the global acetylation of histone H3 by 1.3 times without affecting other modifications. In addition, it was shown that LCS-1269 reduces the expression of mRNA genes of the histone deacetylases HDAC1 and HDAC3 by 1.2 times, and also inhibits the expression of the HDAC1 protein by 1.6 times. The results of methyl-sensitive restriction analysis and ELISA for methylated DNA showed that under the action of LCS-1269, the integral methylation of DNA decreases by 1.4 times.

**Discussion:** Thus, we showed for the first time the ability of the indolocarbazole derivative LCS-1269 to reactivate epigenetically repressed genes. The epigenetic effect of the compound is realized through the mechanisms of histone acetylation and DNA methylation by inhibiting the corresponding enzymes. This work was supported by the RSF (grant no.21-75-10163).

## 2023 ICHAMS Presentations

### Plenary Sessions

#### PS1: In vitro analysis on master transcriptional regulators identifies tissue factor pathway inhibitor 2 as a novel biomarker for ulcerative colitis non-responders

##### Helen Huang^1^, Luke Grant^2^, Ololade Lawal^2^, Sudipto Das^2^

###### ^1^School of Medicine, Royal College of Surgeons in Ireland; ^2^Epigenetics and Gastrointestinal Diseases Research Group (EpiGastroDRG), School of Pharmacy and Biomolecular Sciences, Royal College of Surgeons in Ireland

####### **Correspondence:** Helen Huang

*BMC Proceedings 2023*, **17(Suppl 17):**PS1

**Introduction**: Effective patient management for ulcerative colitis (UC) is hampered by a lack of molecular biomarkers that can predict response to Infliximab. However, preliminary *in silico* results from our lab, for the first time, identified master transcriptional regulators (MTRs) that regulate gene expression underpinning response to Infliximab. The aim of this study was to examine the expression levels of these MTRs using *in vitro* models of UC to further identify biomarkers for Infliximab non-responders.

**Methods**: Normal rectal epithelial cells were cultured and split into three replicates: vehicle (BSA 1.0%), cells only (CO), and cells treated with TNFa (20 ng for 24 hrs) to develop a pro-inflammatory phenotype recapitulating UC. mRNA levels of up-regulated (*TFPI2*, *CXCL8, SELE, FCGR3B)* and down-regulated (*FGFR2)* MTRs previously identified were extracted. A quantitative real-time PCR (qRT-PCR) was carried out to assess levels of MTRs post-TNFa stimulation. Values from qRT-PCR experiments were recorded and fold expressions (R.Q.) were obtained by calculating mRNA expression of MTRs in CO and TNF compared to vehicle. The data was analysed in Microsoft Excel and graphed using PRISM.

**Results**: Higher expressions of *TFPI2* was statistically significant in cell lines treated with TNFa compared to cells without treatment (p=0.03). While *CXCL8* expression was higher in TNFa treated cell lines, the gene did not reach statistical significance when comparing with vehicle treated cells (p=0.051). In comparison, *SELE* and *FCGR3B* expression was not statistically significant. Of the previously identified downregulated MRs, lower expressions of *FGFR2* was statistically significant in cell lines treated with TNFa compared to cells without treatment (p=0.01).

**Discussion**: Our study suggests, for the first time, that *TFPI2* may act as a novel biomarker for IBD treatment response. Future studies should be performed to assess the impact of *TFPI2* knock-down on pro-inflammatory cytokines central to UC development and elucidate its phenotype in non-responders.

#### PS2: Should we monitor cerebral oxygenation in patients undergoing thoracic surgeries?

##### Kajetan Kiełbowski^1^ , Anna Lesińska^2^ , Krzysztof Safranow^3^ , Jarosław Pieróg^1^ , Janusz Wójcik^1^ , Norbert Wójcik^1^ , Małgorzata Wojtyś^1^ , Tomasz Grodzki^1^ , Bartosz Kubisa^1^

###### ^1^ Department of Thoracic Surgery and Transplantation, Pomeranian Medical University, Szczecin, Poland; ^2^ Department of Invasive Cardiology, District Hospital, Szczecin, Poland;^3^ Department of Biochemistry and Medical Chemistry, Pomeranian Medical University, Szczecin, Poland

####### **Correspondence:** Kajetan Kiełbowski

*BMC Proceedings 2023*, **17(Suppl 17):**PS2

**Introduction:** Near-Infrared Spectroscopy (NIRS) is a non-invasive method of regional tissue oxygenation measurement. Intraoperative monitoring of brain oxygenation (BO) can help in identifying cerebral desaturations. We aimed to examine if commonly used peripheral blood saturation monitoring (SvO2) sufficiently represents BO.

**Methods:** SvO2 and BO were measured in a group of 100 patients undergoing standard thoracic surgeries. We have noted the observations every 15 minutes. Statistical analysis was performed using Wilcoxon and Mann-Whitney U tests. Correlations between measured parameters were performed with Spearman’s rank correlation.

**Results:** The duration of included surgeries varied from 30 to 200 minutes. A negative correlation between age and BO at the beginning of surgery was found. A positive correlation between SvO2 and BO between 15 and 90 minutes of surgery was observed. Subsequently, we found a negative correlation between these two methods. SvO2 returned to baseline values faster compared to BO. Nevertheless, both SvO2 and BO showed a negative correlation with overall duration of surgery. Minimum SvO2 values were higher in patients undergoing left-sided procedures. Moreover, significant BO differences were observed in patients undergoing different types of surgery. Wedge resections were associated with higher BO values compared to lobectomies.

**Discussion:** Cerebral desaturation is associated with several risk factors, including one-lung ventilation, hypoxic pulmonary vasoconstriction, and lateral decubitus position. This study shows that SvO2 and BO are not identical. Normal SvO2 measurement does not exclude cerebral desaturation. Furthermore, changes in BO values are associated with patients’ age, duration, and extent of surgery.

**Annotation:** This study is a PhD dissertation of Dr Anna Lesińska

#### PS3: ETV6 Deficiency Unlocks ERG-Dependent Microsatellite Enhancers to Drive Aberrant Gene Activation in B-Lymphoblastic Leukemia

##### Niharika Rajesh^1^, Rohan Kodgule^2^ , Joshua Goldman^2^ , Alexander Monovich^2^ , Travis Saari^2^ , Cody Hall^2^ , Athalee Aguilar^2^ , Juhi Gupta^2^ , Shih-Chun Chu^2^ , Li Ye^2^ , Aishwarya Gurumurthy^2^ , Ashwin Iyer^2^ , Noah Brown^2^ , Mark Chiang^2^ , Martin Cieslik^2^ , Russell Ryan^2^

###### ^1^School of Medicine, Royal College of Surgeons in Ireland; ^2^University of Michigan

####### **Correspondence:** Niharika Rajesh

*BMC Proceedings 2023*, **17(Suppl 17):**PS3

**Introduction:** Distal enhancers play critical roles in sustaining oncogenic gene expression programs. The ETV6-RUNX1 (E-R) fusion oncogene defines a common B-acute lymphoblastic leukemia (B-ALL) subtype, 52 representing about 25% of pediatric B-ALL. However, the mechanism 59 by which ETV6 dysfunction contributes to leukemia is poorly understood. We identify aberrant enhancer-like activation of GGAA tandem repeats as a characteristic feature of B-ALL with genetic defects of the ETV6 transcriptional repressor, including ETV6-RUNX1+ and ETV6-null B-ALL.

**Methods:** A variety of basic and advanced laboratory methods were utilized to obtain our results. These include STR analysis to identify cell lines, cell culture, lentivirus vector generation for ETV6 transgene experiments, FISH analysis, Western blotting, WGS, ChIP-Seq protocols, ATAC-Seq nuclei isolation and library preparation, RNA-seq, gene expression analysis, chromatin data analysis, comparative genomic analysis, CRISPR-interference and Flow cytometry.

**Results:** We show that GGAA repeat enhancers are direct activators of previously identified ETV6-RUNX1+/- like B-ALL “signature” genes, including the likely leukemogenic driver EPOR. When restored to ETV6-deficient B-ALL cells, ETV6 directly binds to GGAA repeat enhancers, represses their acetylation, downregulates adjacent genes, and inhibits B-ALL growth. 1030 out of 1133 enhancer-like GGAA repeat elements in genome-wide analysis were associated with ETV6-bound GGAA repeats (p<0.05). Thus, without intact ETV6 genes, E-R genes are activated in E-R+ B-ALL by the unopposed GGAA repeats. In ETV6-deficient B-ALL cells, we find that the ETS transcription factor ERG directly binds to GGAA microsatellite enhancers and is required for sustained activation of repeat enhancer-activated genes. CRISPRi-mediated ERG knockdown resulted in significantly decreased transcript levels for 36 of 40 genes in the ETV6-repression signature by RNA-Seq, showing its role in B-ALL.

**Discussion/Conclusion:** Together, our findings reveal an epigenetic gatekeeper function of the ETV6 tumor suppressor gene and establish microsatellite enhancers as a key mechanism underlying the unique gene expression program of ETV6-RUNX1+/-like B-ALL.

#### PS4: Moving a suicide bereavement peer-support group online due to COVID-19: the experiences of group facilitators and attendees

##### Niall Seymour^1^ , Selena O' Connell^2^ , Eimear Ruane-Mcateer^2^ , Eve Griffin^2^

###### ^1^ School of Medicine, University College Cork, Cork, Ireland; ^2^ School of Public Health, University College Cork, Cork, Ireland. National Suicide Research Foundation, Cork, Ireland

####### **Correspondence:** Niall Seymour

*BMC Proceedings 2023*, **17(Suppl 17):**PS4

**Background:** Suicide is recognised worldwide as a major public health issue; with a lifetime prevalence of losing a close friend or relative to suicide in excess of 20%. Those bereaved due to suicide experience unique challenges – including being at a higher risk of suicidal ideation, depression, and post-traumatic stress disorder. People bereaved by suicide often turn to peer support groups, facilitated by people who have been similarly bereaved, in an effort to deal with their grief. This study aimed to assess the impact of moving such groups from an in-person to an online format during the COVID-19 pandemic.

**Methods:** The study was carried out with HUGG, an organisation providing suicide bereavement peer-support in Ireland. A mixed methods approach was used. HUGG group attendees were invited to complete an online survey and an online semi-structured focus group was held with HUGG group facilitators.

**Results:** 74% (n=17/23) of survey respondents said they would prefer a blended (in-person and online) or online-only approach to future peer-support groups. Reported benefits of the online format included greater flexibility, not requiring childcare, not having to drive or use public transport after emotional meetings, and being able to leave meetings easily. Drawbacks of the online format included difficulties with providing appropriate support and follow up if a group member became distressed, lack of body language, meetings being more tiring/draining, and the lack of privacy in a home environment. Computer skills did not appear to be an issue, with 87% (n=20/23) of respondents being “extremely confident” at using the computer and internet.

**Discussion:** Group members and facilitators were highly satisfied with participating in online suicide bereavement support groups. Going forward, this study highlights that the online format may continue being of value, particularly for people who are unable or would prefer not to attend in-person support groups.

### Oral Sessions

#### O1: ADAM-17 serum concentration in Alpha-1-antitrypsin deficient patients

##### Domonique Proceviat^1^, Mark Murphy^2^, Malcolm Herron^2^, Emma Leacy^2^, Noel G Mcelvaney^3^

###### ^1^School of Medicine, Royal College of Surgeons in Ireland; ^2^Respiratory Research Division, Department of Medicine, Royal College of Surgeons in Ireland, Education and Research Centre, Beaumont Hospital Dublin, Ireland; ^3^Head Of School Of Medicine, Department of Medicine, Royal College of Surgeons in Ireland

####### **Correspondence:** Domonique Proceviat

*BMC Proceedings 2023*, **17(Suppl 17):**O1

Alpha-1-antitrypsin deficiency (AATD) is a syndrome that predisposes patients to pulmonary emphysema and liver complications. While alpha-1-antitrypsin is often investigated for its antiprotease activity, it also inhibits pro-inflammatory molecules, including ADAM-17. ADAM-17 cleaves cytokines, receptors, and has been implicated in many pulmonary pathologies. Here we measure the differences in ADAM-17 concentration in the serum of patients with different AATD phenotypes. Whole blood was collected from 28 patients of varying phenotypes and processed for serum. ADAM-17 was measured using an R&D DuoSet ELISA kit, and an ELISA was also done to measure IL-6 and sTNFR1 concentration. Clinical and biochemical data were retrieved from the Beaumont Hospital PIPE system. Data was analysed in GraphPad Prism (version 9.4.0). Kruskal-Wallis tests were used for inter-group comparisons, and cytokine levels were correlated with clinical readouts using Pearson Correlation. We found ADAM-17 concentration to be significantly higher levels in ZZ patients compared to MMs (p=0.0225). ADAM-17 was also correlated to IL-6 and sTNFR1 concentration in the serum (p<0.0001 for both). Correlating ADAM-17 with the available pulmonary function tests (PFTs) for the patient cohort, ADAM-17 concentration was positively correlated with DLCO (p=0.0192). With data gathered from the Beaumont Hospital PIPE system, ADAM-17 was found to be positively correlated to serum IgA concentration (p= 0.0028). IL-6 was positively correlated with FEV1 (p=0.0076), FEV1(%) (p=0.0230), FEV1/FVC (p=0.0292), and DLCO (p=0.0174). Due to the significantly higher concentration of ADAM-17 in ZZ patients, this data is suggestive that ADAM-17 may have a role in the pathogenesis of severe AATD. However, since the ADAM-17 concentration shows a paradoxical relationship to PFTs, an activity assay should be performed in addition to measuring how much of the serum ADAM-17 is proteolytically active. It is evident that ADAM-17 is a piece of the puzzle in AATD, and this poses an opportunity to investigate the exact mechanism.

#### O2: Arginase-2 induction is TLR4 specific in inflammatory macrophages

##### Rachel Frankle^1^, Jennifer Dowling^2^, Remsha Afzal^2^

###### ^1^School of Pharmacy and Biomolecular Sciences, Royal College of Surgeons in Ireland; ^2^School of Pharmacy and Biomolecular Sciences, Royal College of Surgeons in Ireland

####### **Correspondence:** Rachel Frankle

*BMC Proceedings 2023*, **17(Suppl 17):**O2

During infection, the toll-like receptor (TLR) family of proteins in macrophages detect unique pathogen- and damage-associated molecular patterns and initiate an immune response accordingly. This response is mediated by differential expression of inflammatory mediators e.g., TNF, IL-1, and IL-6 etc.1 Although macrophages upregulate acute inflammation, they can later switch their functional phenotype to produce anti-inflammatory cytokines, such as IL-10, to resolve inflammation.2 Dysregulation of TLR signalling and macrophage polarisation states are implicated in a diverse range of pathologies, from chronic infection to immunodeficiency.1,3 The enzyme arginase-2 (Arg2) is required for IL-10-mediated polarisation of macrophages to an anti-inflammatory state via downregulation of the inflammatory cytokine IL-1β. Specifically, the IL-10/Arg2 pathway is induced in macrophages stimulated with lipopolysaccharide (LPS), a TLR4 agonist.4 We explore whether other TLR signalling pathways induce Arg2 in the presence of IL-10, and hence which TLR-linked pathologies might benefit from Arg2 activation. We stimulated macrophages for 24 hours with LPS or Pam3CSK4 (TLR1/2 agonist) with and without IL-10 in wild type (WT) and Arg2 knock-out (KO) mice. Gene and protein expression were analysed via RT-PCR and western blotting, respectively. We characterised changes in Arg2 expression, as well as the processing and secretion of IL-1β. Our results demonstrated that whilst IL-1β was upregulated by both TLR1/2 and TLR4, Arg2 was induced more selectively by TLR4, an effect observed at both the mRNA and protein level. Moreover, we confirmed that whilst Arg2 KO macrophages were unable to suppress LPS-induced IL-1β with IL-10 pre-treatment, they retained the ability to downregulate Pam3CSK4-induced IL-1β levels. Collectively, this data shows Arg2 holds promise as a novel anti-inflammatory target specifically in TLR4-mediated signalling. Future work will examine the therapeutic role of Arg2 in models of TLR4-centric diseases, as well as the ability of other TLRs to activate the IL-10/Arg2 axis in macrophages.

#### O3: Brain macrostructure in neonates with hypoxic-ischemic encephalopathy

##### Bhavya Kapoor^1^, Lilian Kebaya^2^, Paula Camila Mayorga^3^, Paige Meyerink^3^, Talal Altamimi^3^, Emily S. Nichols^4^, Sandrine de Ribaupierre^3^, Soume Bhattacharya^3^, Leandro Tristao^5^, Michael T. Jurkiewicz^5^, Emma Duerden^2^

###### ^1^Royal College of Surgeons in Ireland; ^2^Neuroscience, Western University; ^3^Neonatal-Perinatal Medicine, Department of Paediatrics, London Health Sciences Centre; ^4^Applied Psychology, Faculty of Education; ^5^Western University, Department of Medical Imaging, London Health Sciences Centre

####### **Correspondence:** Bhavya Kapoor

*BMC Proceedings 2023*, **17(Suppl 17):**O3

**Background**: Hypoxic ischemic encephalopathy (HIE) is a severe brain injury impacting term-born neonates and is associated with a myriad of adverse developmental outcomes suggesting the involvement of subcortical structures with extensive cortical connections.

**Aims:** 1) To examine subcortical macrostructure in the first few days of life in neonates with HIE compared to age- and sex-matched healthy newborns. 2) To determine whether subcortical volumetric maturation is associated with HIE severity.

**Methods**: A cohort of 28 newborns (19 males [67.9%], median gestational age [GA] =38.6 weeks) with HIE (mild = 4, moderate =21, severe =3 based on Sarnat staging) were scanned with MRI within the first four days of life (median postmenstrual age [PMA]=39.2). The control group included 28 healthy newborns matched for GA, birth weight and PMA at scan. Subcortical volumes (thalamus, basal ganglia, hippocampus, cerebellum) were automatically extracted from T1-weighted images and generalized linear models assessed between-group volumetric differences. Within-group analyses assessed the association amongst subcortical volumes, HIE severity and cooling status.

**Results:** Newborns with HIE had significantly smaller bilateral thalamic, basal ganglia, cerebellar and right hippocampal volumes compared to healthy newborns (all, p<0.001). Newborns with HIE had significantly larger ventricular volumes (all, p<0.001) compared to controls. Greater HIE severity was associated with smaller volumes of the right putamen (p <0.001), left putamen (p<.001), right (p<0.001) and left pallidum (p=0.015) when adjusting for clinical and demographic factors.

**Conclusions:** Newborns with HIE, scanned with MRI within the first days of life, had smaller subcortical volumes impacting sensory and motor regions compared to healthy newborns. HIE severity was associated with smaller volumes, particularly impacting the basal ganglia, suggesting heightened vulnerability of these structures to perinatal asphyxia.

#### O4: Destigmatizing differences—one word at a time: health professions students’ perspectives on stigmatizing language use in healthcare

##### Saakshi Daswani^1^, Elizabeth Gorecki^1^, Jacinta Burke^2^, Lisa Mellon^3^

###### ^1^Graduate Entry Medicine, Royal College of Surgeons in Ireland; ^2^Centre for Mastery: Personal, Professional & Academic Success, RCSI University of Medicine and Health Sciences; ^3^Department of Health Psychology, School of Population Health, RCSI University of Medicine and Health Science

####### **Correspondence:** Saakshi Daswani

*BMC Proceedings 2023*, **17(Suppl 17):**O4

**Background:** The use of stigmatizing words and phrases such as “disorder,” “defect,” “substance-abuser,” “demented,” is not uncommon in healthcare. It influences the perceptions healthcare providers have of their patients and affects care delivery. In an inclusive healthcare environment, the use of person-first language is considered best practice. However, guidelines surrounding this are not formalized and ever-changing.

**Methods:** This qualitative study examined medical students’ perspectives on the use of stigmatizing language in teaching and clinical settings, and recommendations for improvement. 21 medical students at RCSI University of Medicine and Health Sciences participated in four semi-structured focus group interviews. The interviews were transcribed, and two independent reviewers coded the data using thematic analysis. A third reviewer helped resolve any disagreements.

**Results:** Seven key themes (average Kappa coefficient of agreement = 44%) surrounding the use of stigmatizing language in medical education were identified: the prevalence of stigmatising language (“insidious”), its impact on students and patients (“it stops people getting the help they need”), the fine-line between being sensitive vs. medically correct (“do you change scientific lexicon to accommodate trends”), how recommendations for language use is constantly evolving (“it’s hard to keep up”), barriers to change practice (“we don’t have the tools”), how power dynamics and cultural context can influence language use, how this is a broader societal issue (“this isn’t a problem we can solve just in medicine”). Participants further provided recommendations for improvement, including more training, facilitating workshops, and sharing patients’ stories.

**Conclusion:** Study findings provide insight into health professions students’ perspectives on stigmatizing language use in healthcare and recommendations for betterment. This is a continuously evolving topic with little guidance. Results from this study can inform the development of formalized direction to address stigmatizing language use in healthcare, particularly coming from and catering to future generation of healthcare professionals.

#### O5: Identification of cancer driver mutations in chromatin remodelling genes

##### Dahye Shin^1^, Nicola Cosgrove^2^, Simon Furney^2^

###### ^1^School of Medicine, RCSI University of Medicine and Health Sciences, Dublin, Ireland; ^2^Genomic Oncology Research Group, Dept. of Physiology and Medical Physics, RCSI

####### **Correspondence:** Dahye Shin

*BMC Proceedings 2023*, **17(Suppl 17):**O5

Chromatin-remodelling genes are a group of genes coding proteins related to the chromatin rearrangement from a condensed state to a transcriptionally accessible state. These genes affect gene expression epigenetically by modifying histones or acting as transcription cofactors. These chromatin-remodelling gene mutations appear in many types of cancers. This research focused on the most frequently reported colorectal, lung and breast cancer and compared the rate and characteristics of chromatin remodelling gene mutations according to different cancer types. This study is a retrospective cohort study using bioinformatics, and I conducted this research through the R statistics package to analyse cancer cohorts presenting somatic mutations. This study focused on characterising chromatin-remodelling gene mutations among 271 colorectal adenocarcinoma data (COAD), 409 samples of lung adenocarcinoma and 365 samples of breast cancer data by The Cancer Genome Atlas (TCGA). Recurrent somatic mutations are identified in chromatin-remodelling genes in the Colon (COAD), Lung (LUAD) and Breast (BRCA) cancer cohort. The data shows different kinds of mutated chromatin-remodelling genes in each cohort. In COAD cases, KMT2D is the highly mutated gene, followed by KMT2C and KMT2B. PRDM9 and KMT2C are the most frequently mutated genes in LUAD and BRCA, respectively. Overall, KMT2C is one of the most frequently mutated chromatin-remodelling genes. In conclusion, our data indicate that there are common and frequently mutated chromatin remodelling genes despite the variations in somatic mutation types and their affecting loci.

#### O6: Leukodystrophy-linked mutant claudin-11 sensitizes cells to endoplasmic reticulum stress-induced death

##### Caroline Mccamus, Mahmoud Pouladi

###### Department of Medical Genetics, Center for Molecular Medicine and Therapeutics, British Columbia Children's Hospital Research Institute, University of British Columbia

####### **Correspondence:** Caroline Mccamus

*BMC Proceedings 2023*, **17(Suppl 17):**O6

**Introduction/Background:** ER stress-induced cell death is a cellular process that is triggered under a variety of conditions in response to disturb protein folding in the ER. There are several diseases associated with ER-stress induced cell death, including Pelizaeus-Merzbacher disease (PMD). The mechanisms by which ER-stress-induced cell death leads to disease is still unclear. Stoploss mutations in CLDN11, the gene encoding the tight junction protein claudin-11, have recently been identified as a novel cause of PMD. How mutant claudin-11 causes PMD is unclear. In this project, we sought to test the hypothesis that the PMD-linked stoploss mutations induce ER stress as a result of mutant claudin-11 misfolding, sensitizing to ER stress-induced cell death.

**Methods:** HEK293 cells with stable expression of wildtype (WT) or mutant (MT) claudin-11 were assessed by Western blot to examine the impact of the stoploss mutations on claudin-11 expression. The Claudin-11 HEK293 cells were also evaluated using assays of cell viability and death at baseline and following treatment with the ER stress induced Thapsigargin (Tg).

**Results:** Western blot analyses showed that the stoploss mutation causes a shift in MT claudin-11 to a higher molecular weight species and appears to decrease its steady-state levels. Treatment with the ER stressor Tg (3 and 6μM) for 24-48 hrs resulted in lower cell viability and higher cell death in MT claudin-11 HEK293 cells compared with WT.

**Conclusions:** Our results indicate that MT claudin-11 sensitizes cells to ER stress-induced cell death, and suggest that ER stress may contribute to CLDN11-induced leukodystrophy. More broadly, ectopic expression of transgenes in immortalized cells can allow the impact of different mutations to be further examined. This can help clarify the impact that specific mutations have on ER function and how ER stress can lead to cell death in cells harbouring mutant proteins.

#### O7: Testing a novel JAM-A inhibitor derivative for application in targeted drug therapy in breast cancer: a pre-clinical pilot study

##### Sarah Chiodo^1^, Lorna Williams^1^, Lance Hudson^2^, Cathy Richards^3^, Ann M. Hopkins^2^

###### ^1^Medical student, Royal College of Surgeons in Ireland; ^2^Department of Surgery, RCSI University of Medicine and Health Sciences, RCSI Smurfit Building, Beaumont Hospital, Dublin 9, Ireland; ^3^Department of Medicine, RCSI University of Medicine and Health Sciences, RCSI Smurfit Building, Beaumont Hospital, Dublin 9, Ireland

####### **Correspondence:** Sarah Chiodo

*BMC Proceedings 2023*, **17(Suppl 17):**O7

**Introduction:** Junctional Adhesion Molecule-A (JAM-A) is emerging as a target of interest in breast cancer due to its reported capacity to regulate HER2 and HER3 expression, and because its cleavage may be a biomarker of resistance to HER2-targeted therapies in patients. A novel antagonistic peptide (JBS2) was previously shown to exert anti-tumour effects in pre-clinical in vivo breast cancer models (1). As peptides are unstable drugs, we aimed to explore whether a stable D-amino acid derivative of JBS2 (JBS2-D) had superior bioefficacy.

**Methods:** In vitro cell viability assays and in vivo chick embryo xenograft assays were performed in HER2+/JAM-A+ SK-BR-3 breast cancer cells overexpressing JAM-A (SK-J+) or empty vector (SK-EV) following treatment with JBS2 or JBS2-D. In vitro, 1,500 cells/well were seeded onto 96-well plates, treated for 3-6 days with JBS2 or JBS2-D (50-500μg/mL) versus vehicle (10 μL PBS), and subjected to MTT viability assays. In vivo, 2x106 cells were implanted onto the chorioallantoic membranes of fertilised hen eggs, and xenografts harvested following 4-day exposure to JBS2, JBS2-D (250μg/egg) or vehicle (PBS 25μL/egg).

**Results:** JBS2 and JBS2-D exerted concentration- and time-dependent reductions in in vitro cell viability, with the greatest sensitivity to JBS2-D in SK-J+ cells. In the in vivo pilot study, four embryos with grossly-visible tumours survived (vehicle; JBS2; 2xJBS2-D).

**Discussion/Conclusion:** Early indications support the hypothesis that JBS2-D exerts greater bioefficacy in breast cancer models. Results provide a compelling rationale for replicating this pilot study, correcting for design flaws and low sample size.

**Acknowledgements**

We thank the Health Research Board for funding (grant SS-2022-024 to SC), and the RCSI Research Summer School.

### Poster Sessions

#### P1: “Visual snow – systematic review on etiology and treatment.”

##### Wiktoria Stańska, Anna Torbus, Przemysław Rusztyn, Piotr Maciejewicz

###### Department of Ophthalmology, Medical University of Warsaw

####### **Correspondence:** Wiktoria Stańska

*BMC Proceedings 2023*, **17(Suppl 17):**P1

**Introduction:** Visual snow (VS) is a rare clinical entity, described as the bilateral presence of dynamic, flickering dots affecting the visual field, often compared by patients to snow or pixelated television static. It can be a lifelong condition or have an acute onset. Moreover, it is a relatively new term in medicine, and the nature of the condition is subjective and elusive, lowering the quality of life. Unfortunately, little is known about VS. This systematic review aims to describe the updates in the etiology and treatment of visual snow.

**Materials & Methods:** We searched for articles in English, presenting original data and published after December 2019. We also aim to increase awareness of visual snow because many healthcare professionals have difficulty diagnosing it quickly.

**Results:** Different studies show inconsistent data. Neuroimaging found, among others, hypermetabolism of the lingual gyrus, changes in the occipital cortex, increased gray matter in different brain areas, and altered connectivity in visual pathways. However, these findings are not present in all patients. Interestingly, they often do not have abnormalities in the ophthalmic examination. According to the literature, among the most effective drugs are lamotrigine and topiramate. Unfortunately, they also carry a risk of worsening the symptoms. It's crucial to remember that visual snow can be worsened or induced by alcohol, recreational drugs, and particular medication like antidepressants. In terms of treatment, nonpharmacological approaches like color filters and repetitive Transcranial Magnetic Stimulation are also made.

**Discussion:** Further studies are needed to fully understand VS's nature, as current data is based mainly on case reports. Nevertheless, expanding the knowledge about visual snow can impact patients' comfort. That is why hearing what the symptoms are is an immense relief for a patient. As well as a quick and proper diagnosis lets patients avoid stress and shortens the diagnostic path.

#### P2: A novel prognostic scoring system combining the revised Tokuhashi Score and the New England Spinal Metastasis Score for preoperative evaluation of spinal metastases

##### Dionisia Mavritsakis^1^, Louis-Philippe Amiot^2^

###### ^1^Royal College of Surgeons in Ireland; ^2^The Department of Orthopedic Surgery-Spine, Hôpital Maisonneuve-Rosemont

####### **Correspondence:** Dionisia Mavritsakis

*BMC Proceedings 2023*, **17(Suppl 17):**P2

**Introduction:** Numerous scoring systems have been developed in order to determine the prognosis of spinal metastases. Predicting as accurately as possible the life expectancy of patients with spinal metastatic disease is very important, as it is the decisive factor in selecting the most optimal treatment for the patient. The Revised Tokuhashi score (RTS) and the New England Spinal Metastasis score (NESMS) are popular scoring systems used to determine the optimal treatment modality. However, they sometimes provide conflicting results. We propose a novel prognostic scoring system, which combines the (RTS) and the (NESMS) to predict with greater accuracy the prognosis.

**Methods:** We retrospectively reviewed the data of 64 patients with spinal metastasis enrolled between 2012 and 2021 in the Department of Orthopedic Surgery-Spine, Hôpital Maisonneuve-Rosemont, Montréal, Que. The new score per patient was then calculated as a combination of the RTS of each patient and the patient’s corresponding NESMS score and then compared to the actual patient survival period in order to assess its adequacy in predicting the survival of patients with spinal metastases. The patients were divided into three groups: Low, Moderate or Good Prognosis.

**Results:** In the Low Prognosis group, the reliability of predicting the prognosis was 55.6% in 27 patients. In the Moderate Prognosis group, the reliability of predicting the prognosis was 95.8% in 24 patients. In the Good Prognosis group, the reliability of predicting the prognosis was 100% in 13 patients.

**Discussion:** This study demonstrates that a new prognostic scoring system, which would combine the RTS and the NESMS, is promising in providing an improved accuracy for predicting the actual patient survival especially for the moderate and good prognosis patients. An appropriate prospective investigation with a larger sample size should be conducted to further investigate the validity of this novel scoring system and its overall predictive value.

#### P3: A review of international approaches to HTA: how do mechanisms of stakeholder engagement and research dissemination differ?

##### Abubakr El Sheikh Idris^1^, Barbara Clyne^2^, Karen Jordan^3^, Máirín Ryan^3^

###### ^1^Royal College of Surgeons in Ireland; ^2^Department of General Practice RCSI University of Medicine and Health Sciences; ^3^Health Information and Quality Authority, Dublin, Ireland

####### **Correspondence:** Abubakr El Sheikh Idris

*BMC Proceedings 2023*, **17(Suppl 17):**P3

**Introduction:** Health technology assessment (HTA) is research process that supports policymakers in making evidence-based decisions on how best to allocate finite resources within a healthcare system. Previous studies have highlighted the importance of increasing the participation of stakeholders, including patients and the public, in health services research and HTA. However, differences in stakeholder needs often leads to variation in processes between countries. As such, the aim of this study was to outline the processes of international HTA organisations regarding stakeholder engagement and research dissemination.

**Methods:** A mapping review was undertaken comprising a grey literature search of websites of a sample of 14 international HTA organisations. Organisations were selected based on relevance to the Irish context, similar activity and similar ranking on the human development index.

**Results:** In terms of stakeholder engagement, half of the agencies assessed used full public consultations in the preparation of HTAs, whilst 57% (8/14) accepted public comments on a HTA. Five agencies (36%) reported conducting interviews and focus groups to collect input from stakeholders. Almost all agencies (13/14, 93%) involved advisory groups in the production of their HTAs. In all agencies the findings of assessments were made publicly available through a variety of means. Notably, whilst all 14 agencies included summaries within their reports and most (12/14, 86%) also published separate summary documents, only two agencies (14%) published infographics alongside reports.

**Conclusion:** Of the sample of agencies examined, there was variation in the types of stakeholder engagement and modes of dissemination used. Differences of approach may reflect differences in processes and roles between agencies. Measuring the impact of these differences on the outcomes of HTAs is difficult as multiple factors can influence policy and decision-making. Further research is needed to understand the contribution of stakeholder engagement and research dissemination to the impact of HTA research.

#### P4: A Systematic review of blood donation in insulin dependent diabetes mellitus patients

##### Shreyansh Shreyansh^1^, Shivam Pradipkumar Thaker^1^, Vidya Monappa^2^, Shivananda K Bhat^2^

###### ^1^Kasturba Medical College, MAHE, Manipal; ^2^Department of Pathology, Kasturba Medical College, MAHE, Manipal

####### **Correspondence:** Shreyansh Shreyansh

*BMC Proceedings 2023*, **17(Suppl 17):**P4

**Introduction:** Diabetes is a disease that is studied in several fields of clinical practice. In this paper, we present studies of diabetes in relevance to the field of blood transfusion, specifically about donation from insulin-dependent diabetics. The objective of this paper is to interpret relevant information and data pertaining to guiding national healthcare policies that are currently excessively restrictive.

**Method:** Literature for this paper was obtained from PubMed, Embase, and ClinicalKey. Data on Vasovagal reactions from Diabetic Donors were analyzed. Guidelines were obtained from the official websites of the countries included in the review. Data interpretation, analysis, and policies of relevance were assessed by 2 reviewers and entered into the result table.

**Result:** In the search of the literture on this topic, only fifteen papers were found, the majority of which indicated the practice of insulin-dependent diabetics donating blood as being safe, however, our research identified that HbA1c values drop for upto 3 months after donating blood and thus may put such donor at risk of a false indication of glycemic control. Additionally, blood donation leads to reduction in serum iron levels, which in-turn reduces reactive oxygen groups and increased insulin sensitivity, though there is literature to prove otherwise, hence left inconclusive. A vasovagal reaction rate of 4.8% was observed, which was not a significant increase from reaction rate of normal donors. The current status of many national guidelines banning blood donation by insulin-dependent diabetics is unduly conservative. It may be attributed to impact of research on bovine-derived insulin in opposition to the modern rDNA insulin.

**Conclusion:** As per the review of existing research and evidence, authors recommend that it is safe for insulin-dependent diabetics to donate blood as long as they are aware of the impact on their own serum HbA1c values as a result of the donation.

#### P5: A systematic review of international health policy guiding the identification, analysis, and management of genomic secondary findings

##### Safa Majeed^1^, Christine Johnston^1^, Saumeh Saeedi^2^, Chloe Mighton^3^, Vanessa Rokoszak^3^, Sonya Grewal^3^, Vernie Aguda^3^, David Malkin^4^, Yvonne Bombard^3^

###### ^1^Temerty Faculty of Medicine, University of Toronto, Toronto, ON, Canada; ^2^Royal College of Surgeons in Ireland; ^3^Genomics Health Services Research Program, Li Ka Shing Knowledge Institute, St. Michael’s Hospital, Unity Health Toronto, Toronto, ON, Canada; ^4^Division of Hematology/Oncology, The Hospital for Sick Children, Toronto, ON, Canada

####### **Correspondence:** Saumeh Saeedi

*BMC Proceedings 2023*, **17(Suppl 17):**P5

**Introduction:** An explosion in genomic sequencing for precision medicine has yielded an analogous abundance of genetic secondary findings (SFs). SFs can be defined as gene variants identified by genomic sequencing that have potential medical value, but were found incidentally, and are unrelated to the patient's primary reason for testing. These results have the potential to inform patients about their risk of developing a health condition and may result in early screening or preventative measures. Current SF identification, analysis, and management practices are inconsistent and sometimes contradictory, leading to inconsistent patient care and outcomes. As the frequency of genomic profiling rises, a synthesis of the international SF health policy landscape is imperative to understand needs and limitations.

**Methods:** We carried out a systematic review to appraise guidance established internationally directing identification, analysis, and management of SFs for participants receiving genomic sequencing. A comprehensive search in MEDLINE, Embase, and Cochrane databases was conducted. Articles were reviewed using Covidence. Results: We identified 1,028 records and found 63 studies for inclusion producing guidance on SFs based on our eligibility criteria. Most policies focus on SF management (98%; n = 62), but fewer guide bioinformatic analyses (60%; n =38) or identification (48%; n=30). The most frequent topic mentioned was informed consent (56%; n =35) during pre-test management. Lab policies (11%; n =7) including guidance for avoiding SF discovery when requested (11%; n =7) were minimal. Low levels of quality were awarded for evidence used, stakeholder involvement, and applicability for most studies.

**Conclusions:** Our results highlight gaps in policy for SF bioinformatic analysis and identification. Although some management processes are described, policy for medical care following results disclosure remain unknown. Lastly, many studies did not have sufficient evidence to base their guidance on. Future work should fill policy gaps and support evidence-based practice.

#### P6: Assessing the Impact of COVID-19 on Health Resource Utilisation by Paediatric Patients with Cerebral Palsy

##### Natalie South^1^, Sophia Provenzano^2^, Emily Schaeffer^3^, Stacey Miller^4^, Maria Juricic^4^, Kishore Mulpuri^3^

###### ^1^Royal College of Surgeons in Ireland; ^2^University of British Columbia, Vancouver, British Columbia, Canada; ^3^Department of Orthopaedic Surgery, BC Children's Hospital, University of British Columbia, Vancouver, BC Canada; ^4^BC Children's Hospital, Department of Physical Therapy, University of British Columbia, Vancouver, BC, Canada

####### **Correspondence:** Natalie South

*BMC Proceedings 2023*, **17(Suppl 17):**P6

**Introduction:** Cerebral Palsy (CP) is the most common cause of motor impairment or disability in children. One in 400 individuals in Canada have CP and require the continuous support of healthcare professionals. Prior to the start of the COVID-19 pandemic the BC Children’s Hospital Orthopaedic Clinic Cerebral Palsy team conducted a study on the accessibility of care for patients with CP and how it varies across the Gross Motor Function Classification System (GMFCS) levels. The purpose of this cross sectional study is to develop and administer a follow-up survey to assess how access and utilisation of healthcare services by paediatric patients with cerebral palsy has been impacted by the COVID-19 pandemic and to determine any required changes to service delivery.

**Methods:** The original survey used in 2019 was adapted to collect new data on how access to care has changed for CP patients throughout the pandemic. This anonymous survey will be completed by consenting CP patients who attend the orthopaedic clinic at BC Children’s Hospital.

**Results:** Thirteen patients have completed the study to date with surveys still being administered. Based on results to date and anecdotal evidence, CP patients saw a decrease in the frequency of in-person healthcare visits during the pandemic, specifically rehabilitation services.

**Discussion:** The pandemic has had long lasting effects on the delivery of healthcare and this survey allows us to see the direct impact this has had on patients with CP. The overall goal of the study is to identify the gaps in care sustained by patients with CP during the pandemic. This will allow identification of service area needs and adjustments required in resource delivery to accommodate patient needs in the event of future pandemics. This study will also give us insight into the evolving balance of virtual and in-person care.

#### P7: Assessing the patient perspective in a neurovascular clinic: a quality improvement project

##### Minatoullah Habaka^1^, Vitor Mendes Pereir^2^, Nicole Cancelliere^3^

###### ^1^Royal College of Surgeons in Ireland; ^2^Neurosurgical and Medical Imaging, St. Michael’s Hospital; ^3^RADIS Lab, Li Ka Shing Knowledge Institute

####### **Correspondence:** Minatoullah Habaka

*BMC Proceedings 2023*, **17(Suppl 17):**P7

**Introduction:** In recent years, patient-cantered care has become a crucial consideration in the healthcare system. Understanding the patient perspectives allows providers to accurately assess healthcare quality delivery. Medical information has shifted towards developing patient-centred care to ensure high quality care. The two overarching measures under patient-centred care are patient satisfaction and patient experience. Patient satisfaction provides insight on a patient’s perspective with the overall care, whereas patient experience is concerned with a patient’s interactions with the healthcare facility, staff and waiting times. This study aims to identify points of dissatisfaction and ensure patients are receiving high quality care.

**Methods:** Patient experience surveys were administered to all patients being followed up by physicians at the Neurovascular Clinic. The survey measured patient experiences with initial appointments, hospital stays (if applicable), and follow-up appointments. Satisfaction ratings were done on a Likert scale of 1-5 or 1-7, with the higher ends of the scale representing satisfaction, the middle being neutral, and the lower ends of the scale representing dissatisfaction. Surveys were administered to 202 patients over the span of 6 weeks.

**Results:** 108 responses from patients were recorded. Of 108 patients, 73% were female, and 27% were male. A major area of dissatisfaction was waiting room times. Of 101 patients, 49% were satisfied, 32% unsatisfied, and 19% neutral. Major areas which patients expressed satisfaction included patient-healthcare team communication. Of 102 patients, 71% expressed satisfaction with patient-doctor communication, and 70% expressed satisfaction with patient-hospital staff communication. Of 90 patients, 73% expressed satisfaction with patient-nurse communication.

**Discussion:** The patient experience surveys were beneficial in collecting patient feedback and identifying areas of satisfaction and dissatisfaction. Future directions include a phase 2 where applicable changes can be made in the Neurovascular Clinic to improve patient experience and further assessment of patient satisfaction.

#### P8: Assessment of miRNAs as a novel class of therapeutics in neuroblastoma: a systematic review

##### Rama Alkhaldi^1^, Olga Piskareva^2^

###### ^1^School of Medicine, Royal College of Surgeons in Ireland; ^2^Cancer Bio-Engineering Group, Department of Anatomy and Regenerative Medicine, RCSI University of Medicine and Health Sciences, D02 YN77, Dublin, Ireland

####### **Correspondence:** Alkhaldi

*BMC Proceedings 2023*, **17(Suppl 17):**P8

Neuroblastoma is an aggressive paediatric cancer capable of regressing spontaneously and metastasizing to various regions of the body. Current treatment modalities remain insufficient to treat patients with metastatic spread resulting in the malignancy to account for approximately 15% of all paediatric cancer deaths. MiRNAs are endogenous small non-coding RNAs (20-22 nt) that play a role in the tumorigenesis of different cancers including neuroblastoma. They pose as a novel therapeutic agent due to their ability to regulate the expression of various genes of different pathways, causing reduction in tumour mass.

A systematic search of PubMed Database, Google Scholar, and Clinicaltrials.gov were carried out from time of inception until July 20, 2021 using Preferred Adopting Items for Systematic Reviews and Meta-Analyses (PRISMA) guidelines. Keywords included in the search were “miRNA”, “neuroblastoma”, “therapeutics”, “3D”, “mouse”, “in vivo”, “mice,” and “murine”. Only free English full text articles, paid articles accessible for free through RCSI, peer-reviewed articles, clinical trials, in vivo, and 3D in vitro studies were included.

After careful evaluation, 42 studies were included in the qualitative synthesis. We identified a plethora of miRNAs that exert their oncogenic or tumour-suppressive role by targeting various genetic pathways. The inhibition of oncogenic miRNAs by antagomirs or the replacement of tumour suppressive miRNAs poses as a novel method of treating neuroblastoma. This study also assessed the therapeutic efficacy of certain miRNAs such as miR-34a and miR-542-3p via different modes of delivery such as through nanoparticles and viruses. These methods demonstrated promising tumour suppressive function.

Even though there is a myriad of research conducted about the role of miRNAs in neuroblastoma, very few have been validated in 3D in vitro and/or in vivo. Additional pre-clinical studies assessing the therapeutic efficacy of miRNAs in validated settings is mandatory to allow for clinical large-scale trials to be conducted.

#### P9: Biomarker signatures for biomarker-driven targeted therapies in Epilepsy – Progress and Challenges

##### Siddiqah Shah^1^, Shona Pfeiffer^2^

###### ^1^Royal College of Surgeons in Ireland; ^2^Department of Physiology and Medical Physics, Royal College of surgeons in Ireland (RCSI), Dublin, Ireland

####### **Correspondence:** Siddiqah Shah

*BMC Proceedings 2023*, **17(Suppl 17):**P9

Epilepsy is a complex disorder characterized by recurrent seizures with altered brain function. It affects more than 50 million people worldwide and can be caused by genetic defects or acquired through brain insults. This can lead to a cascaded process called epileptogenesis, where studies are aimed at finding biomarkers that target and interrupt these processes. The aim of this systematic review is to determine from previous studies how identification of biomarkers has aided in the progress of epileptic treatment as well as some challenges faced in the process.

Using a comprehensive literature review search on sites such as Embase, MEDLINE (PubMed), Scopus, and Google Scholar, a systematic search was conducted. A MeSH search was formed using key words such as “epilepsy” AND “biomarkers” AND “targeted-therapies” which generated filtered previous studies containing the most recent randomized trials of the topic at hand between the years 2016-2022.

Recent studies have focused on biomarkers such as %REM sleep, DNA Methylation, MicroRNAs and EEG biomarkers. Their established link to epilepsy shows promise in the development of new ways of approach for targeted therapy. Nevertheless, there are challenges surrounding the lack of availability of diagnostic and prognostic biomarkers in the clinical setting due to: applicability and specificity of biomarker signatures to animal models used; heterogeneity of the epilepsies; accessibility of biomarkers; and identification of patterns in the analysis and management of complex data sets using computational modelling. Due to these challenges, treatment for epileptic patients is limited and a cure is still undiscovered.

In conclusion, despite all the current approaches in establishing targeted therapies in epilepsy, the best biomarker is still the first seizure. There is however increasing potential for the discovery of new biomarkers with technological advancements, for instance in using IPSCs (induced pluripotent stem cells) or developing computational models in the detection of biomarkers.

#### P10: Can the uptake of Home Haemodialysis be improved with the utilization of a mobile device application (app) that contains a remote patient monitoring platform in the Irish public healthcare?

##### Danya James, Sherlissa Ali-Thompson, Imogene Mohammed, Amyrathul Munyra MohamedHassan, Shubhi Hamilton, John Keaney, Michael O'Connor, Lina Adil, Jay Hanoudi, Noel Lum, Vicki Sandys, Nurul Jasmine Binti Abd. Shukor

###### Royal College of Surgeons in Ireland

####### **Correspondence:** Danya James

*BMC Proceedings 2023*, **17(Suppl 17):**P10

**Introduction:** The prevalence of end stage renal disease (ESRD) in Ireland is rising, with over 500 new patients developing renal failure yearly. The current renal replacement therapy (RRT) options for ESRD include peritoneal dialysis, hemodialysis, kidney transplantation and conservative management. In-centre dialysis is time-consuming to patients and has significant transport and nursing costs. While there are many apps for patients undergoing peritoneal dialysis or transplant, there is a market for HHD apps in Ireland. Despite the advantages of HHD, uptake is minimal. Medical apps have been shown to increase patient confidence and education about home-based interventions. We sought to obtain the perspectives of CKD patients and clinicians on if the utilisation of a mobile application containing a remote patient monitoring platform would increase the uptake of home hemodialysis.

**Methods:** We conducted semi-structured interviews with 10 patients on dialysis, 5 nurses at the Beaumont Hospital’s Renal Dialysis Clinic. The responses were analysed using thematic analysis.

**Results:** The findings were separated into (1) patient perspectives; (2) clinician perspectives; and (3) opinions on what app components would increase usage. Patients rated medication reminders, a hospital alert button and HHD troubleshooting videos as essential app components that would increase HHD uptake. Clinicians rated remote patient monitoring as essential. Both patients and clinicians stated that in-person interaction and disabilities would still be hindrances to HHD.

**Discussion:** Patient and clinician responses highlighted that a mobile app would increase the uptake of HHD. Similar technology, utilising a remote patient monitoring platform has been successfully employed in home peritoneal dialysis. The success of an HHD app would precipitate reduced costs of in-centre programs and transport on the Irish Healthcare system. Further research can be conducted, and data collected can be used in the production of an app prototype.

#### P11: Central Nervous System Alterations in Primary Sjögren’s Sydrome: an MRI Study

##### László Módis^1^, Zsófia Aradi^2^, Sándor Csaba Aranyi^3^, Tamás Papp^4^, Miklós Emri^3^, Antónia Szántó^2^, Antal Miklós Bugán^1^

###### ^1^University of Debrecen, Faculty of General Medicine, Department of Behavioural Sciences; ^2^University of Debrecen, Faculty of General Medicine, Department of Internal Medicine, Division of Clinical Immunology; ^3^University of Debrecen, Faculty of General Medicine, Department of Medical Imaging, Department of Nuclear Medicine and Translational Imaging; ^4^University of Debrecen, Faculty of General Medicine, Department of Medical Imaging, Division of Radiology and Imaging Science

####### **Correspondence:** László Módis

*BMC Proceedings 2023*, **17(Suppl 17):**P11

**Introduction:** Central Nervous System (CNS) alterations have been observed in magnetic resonance imaging (MRI) studies of primary Sjögren’s syndrome (pSS), however, they are not characterized unequivocally. We examined pSS patients’ cranial MRI to establish how the disease impacts the CNS.

**Methods:** The examination happened through retrospective database-analysis, using the database of the Outpatient Clinic of the Building “C”, Internal Medicine Clinic, Clinical Centre, University of Debrecen. The inclusion criteria were the already-performed cranial MRI and the absence of systematic autoimmune disorders other than pSS. Out of 729 patients registered in the database, 22 patients fulfilled the criteria. A healthy control group was also established. Comparisons were performed between the pSS and the control groups and between patients with and without detectable disease activity. Morphometric analysis of each brain region and asymmetry indices of the nuclei were involved in the study alongside with the patients’ immunological parameters. The statistical analysis happened through T-tests.

**Results:** We summarize the significant results remaining after false discovery rate (FDR) correction. Regarding the morphometric analysis, the right straight gyrus differed significantly between the groups with and without detectable disease activity (p<0.05). There was a significant correlation between the serum level of complement C3 and the volume of the left insula (p=0.01). The asymmetry indices of each nucleus of the thalamus and the entire thalamus correlate with the serum level of complement C4 (p<0.05) and haemoglobin (p=0.01).

**Discussion:** CNS alterations in pSS may be related to the immunopathology due to the aforesaid correlations. Hence, neurology and neuropsychology should have bigger role in the treatment of pSS

#### P12: Community-Based Participatory Research approach: a case study within ‘The Smokeless Village Project’ in Malawi

##### Eunice Phillip^1^, Farah Elnakoury^2^, Joella Simon^1^, Aisling Walsh^1^, Sarah Jewitt^3^, Debbi Stanistreet^1^

###### ^1^School of Population Health, RCSI, Dublin; ^2^School of Medicine, Royal College of Surgeons in Ireland; ^3^Faculty of Social Sciences, UoN, Nottingham, UK

####### **Correspondence:** Farah Elnakoury; Joella Simon

*BMC Proceedings 2023*, **17(Suppl 17):**P12

The tenet of the Community Based Participatory Research (CBPR) approach is to allow the equitable collaboration between stakeholders, community members and researchers through co-creation of knowledge and mutual respect, to improve health outcomes [1, 2]. The aim is to reduce the power imbalance associated with research projects especially in low-resourced communities. Despite this, there is a dearth of information on the perspective of the community and research team on CBPR, as well as the impact of CBPR constructs on capacity building. The Smokeless Village Project (TSVP) aims to reduce household and ambient air pollution through multi-level interventions using a community-led approach.

To design the CBPR approach, within TSVP, a qualitative case study was undertaken. Fourteen key stakeholder semi-structured interviews were conducted with 5 community members, 2 policy makers and non-governmental organisations, and 7 field-based researchers. A deductive and inductive approach to framework analysis was used to evaluate the interviews. Matrixes of generated codes were done in NVivo 12 software. Six main themes were identified as essential to CBPR. These are co-creation of knowledge, community and research dynamics, conflicts, evaluation of participatory approach, mobilization of community resources, and participation. Findings were incorporated into the TSVP processes which resulted in improved communication, enhanced engagement, and total community buy-in of the project interventions.

The high value allotted by the community members to the processes of open dialogue, research team engagement in community activities, skill acquisition, and respect for culture , norms, and community members, highlighted the importance of community involvement in project success.

#### P13: Comparison of Regurgitant Volumes in Patients with Mitral Valve Insufficiency using 4D Flow MRI and Transthoracic Echocardiography

##### Adarsh Aratikatla, Michael Markl, Taimur Safder, Gloria Ayuba, James Thomas, Jeesoo Lee

###### Northwestern University

####### **Correspondence:** Adarsh Aratikatla

*BMC Proceedings 2023*, **17(Suppl 17):**P13

**Background:** Regurgitant volume (RVol) is a key metric used to determine mitral valvular regurgitation (MVR) severity [1]. Transthoracic echocardiography (TTE) and an indirect approach using cardiac MRI, are both traditional modalities used to quantify RVol but they comprise of limitations affecting its precision and reproducibility, along with error amplification when cardiac abnormalities present [1-3]. 4D-flow-eMRI is a promising technique for MVR flow quantification, since it retrospectively places a plane directly across the flow jet to quantify RVol.

**Methods**: 4D-flow-MRI was performed on 22 patients diagnosed with MVR based on them also having received an Echo within 3 months prior to the MRI. Echo RVol was measured via the PISA method, whereas 4D flow RVol was measured at 7 equidistant planes, placed along the MVR jet [1].

**Results:** 4D flow MRI-RVol and Echo-RVol values were 21.5±17.0ml and 36.0±26.0ml (p<0.005), respectively. All correlation values between 4D flow MRI-RVol and Echo-RVol were >0.75 at every plane. Bias between 4D flow MRI and TTE reduced as the plane was moved downstream, but regardless of plane location (PL), limits of agreement tended to stay similar. TTE overestimated 4D flow MRI measurements at every plane.

**Discussion:** As expected, a consistent increase in 4D flow RVol was observed as the plane moved downstream. However, regardless of where the PL was on 4D flow MRI, TTE still tends to consistently overestimate RVol. These results lead us to believe that patients are vulnerable to being diagnosed with a more severe case of MVR than they truly have; and since this severity guides clinicians to recommend surgery, patients may be undergoing premature or unnecessary interventions. Further studies are warranted to determine whether 4D flow MRI is underestimating, or TTE is overestimating, the true RVol. Subsequently, in vitro models can be used to compare both imaging modalities with the ground truth.

#### P14: Cortisol secretion in psychosis: a systematic review and meta-analysis

##### Henna Elahi^1^, Mary Cannon^1^, Darren Roddy^2^

###### ^1^Royal College of Surgeons in Ireland; ^2^The School of Medicine, Trinity College Dublin, The University of Dublin

####### **Correspondence:** Henna Elahi

*BMC Proceedings 2023*, **17(Suppl 17):**P14

**Introduction:** One of the many roles of the hypothalamic-pituitary-adrenal axis (HPA) is to mediate the physiological response of the human body to stressful stimuli. The hormone end-product of this axis is cortisol. In recent literature, aberrant functioning of this axis and altered levels of cortisol have been theorised to be linked to the pathogenesis of psychotic disorders including schizophrenia. However, current evidence has not yet been able to prove a definite relationship between psychotic disorders and abnormal cortisol levels.

**Methods:** Current studies of cortisol levels in patients with psychosis were collected by a literature search of Pubmed and Google Scholar. Data extraction was carried out on all relevant articles which evaluated cortisol levels throughout the day in patients with schizophrenia, schizoaffective disorder, and first-episode psychosis. Studies that reported morning cortisol values, such as the cortisol awakening response (CAR) were sorted for inclusion and analysis. Data, including the mean cortisol concentration, standard deviation, and p-value, was then stratified and analysed by the type of sample obtained including plasma, salivary, and cerebrospinal fluid (CSF) cortisol.

**Results:** A total of 20 studies were included and analysed for the association between CAR and psychosis. These yielded a moderate effect size with a Cohen’s d-value of +0.46. However, these studies also exhibited marked heterogeneity with an I2 value of 94%.

**Discussion:** Though the included studies showed a positive association between awakening cortisol levels and psychosis, high heterogeneity between the studies limits the ability to establish a clear association. The marked heterogeneity may suggest a lack of association between altered cortisol levels and psychosis, however, further research - perhaps with larger samples - is required to confirm whether there is a true association.

#### P15: Critical shortage of capacity to deliver safe paediatric surgery in Sub-Saharan Africa: evidence from 67 hospitals in Malawi, Zambia, and Tanzania

##### Muskan Sardana, Jakub Gajewski, Chiara Pittalis

###### Royal College of Surgeons in Ireland

####### **Correspondence:** Muskan Sardana

*BMC Proceedings 2023*, **17(Suppl 17):**P15

**Introduction**: Paediatric disease is rife in Sub-Saharan Africa (SSA). Approximately 85% of children within SSA are expected to require surgical intervention by 15, yet barely a fraction receive adequate care. SSA is a young region with 42% of its inhabitants under 15 years of age. Hence, global efforts in upscaling paediatric surgical capacity to meet the continuously growing demand should be a priority.

**Methods:** Data from 67 district-level hospitals in Malawi, Tanzania, and Zambia (MTZ) were collected and analysed using the PediPIPES survey. The five components of PediPIPES are personnel, infrastructure, procedures, equipment, and supplies. A PediPIPES Index was calculated for each country, and a 2-tailed variance test (ANOVA) analysis was used to explore cross-country comparisons, using Jamovi v2.3.18.0.

**Results:** Major personnel shortages observed; no paediatric/general surgeons and anaesthesiologists available in any of the hospitals. Infrastructural capacity not adequate; no hospitals in Malawi had uninterrupted access to external electricity. Paediatric-sized supplies (chest tubes and urinary catheters) not available in 87% of all hospitals. Common surgery, such as open treatment of fracture, least observed in Zambian hospitals (23%). Repair of spina bifida observed in no hospital. No significant difference found in paediatric surgical capacity between MTZ.

**Conclusions:** The lack of specialist personnel available is fundamental to the shortcomings of paediatric surgery in SSA. Local training programs on common paediatric procedures and international medical partnerships must be implemented by ministries to combat shortages. Securing investment in basic healthcare infrastructure, paediatric equipment, and supplies is equally paramount to upscaling safe paediatric surgery in SSA.

#### P16: Cutting Carbs to Combat Conditions: The Effect of a Low-Carbohydrate Diet on Alzheimer’s Disease & Parkinson’s Disease: A Systematic Review

##### Gina Rizq^1^, Samuel Gobraeil^2^, Jala Rizq^3^

###### ^1^Royal College of Surgeons in Ireland; ^2^University of Guelph; ^3^Western University

####### **Correspondence:** Gina Rizq

*BMC Proceedings 2023*, **17(Suppl 17):**P16

**Introduction:** Neurocognitive diseases (NCD) are neurological disorders that cause the deterioration of cognitive functions and ultimately, a significant determinant in one’s quality of life. Numbers have been increasing at alarming rates. Researchers found that dementia patients are doubling every 20 years with cases expecting to reach up to 115 million worldwide by 2050. Likewise, the search for prevention and treatments for NCDs has correspondingly increased with the gravity of the situation.

**Methods**: Using the databases PubMed and the Cochrane Library, a systematic search was conducted and results were narrowed to compiling (7) articles. A MeSH search was formulated with key words such as “Parkinson’s disease & Alzheimer’s disease” and “Ketogenic diet/low carb diet”, and the results filtered to include randomized control trials between the years 2005-2022.

**Results:** It was found that there is ample evidence suggesting that low-carbohydrate diets may have a positive impact on both the onset and progression of various neurological disorders. This includes the implementation of a ketogenic diet as a means of prevention and treatment of Alzheimer’s Disease (AD). It was also found that dietary ketosis plays a role in memory enhancement and positively impacts cognitive impairment in Parkinson’s Disease (PD).

**Discussion:** Overall, low-carbohydrate diets are described as being a “non-pharmacological treatment” with regards to certain disorders, and have proven to play a significant role in major neurological diseases.

#### P17: Deeper phenotyping of TM6SF2 to characterize hepatic steatosis, lipid traits, and metabolic risk factors using a genome-first approach

##### Helen Huang^1^, Carolin Victoria Schneider^2^, Daniel J. Rader^2^

###### ^1^School of Medicine, RCSI University of Medicine and Health Sciences, Dublin, Ireland; ^2^Division of Translational Medicine and Human Genetics, The Perelman School of Medicine, University of Pennsylvania

####### **Correspondence:** Helen Huang

*BMC Proceedings 2023*, **17(Suppl 17):**P17

I**ntroduction:** Using a genome-first approach, we performed deeper phenotyping of steatosis-associated variants in TM6SF2 to better elucidate its prevalence in non-alcoholic fatty liver disease (NAFLD), non-alcoholic steatohepatitis (NASH), and hepatocellular cancer (HCC), while expanding its associations to metabolic and lipid phenotypes.

**Methods:** We leveraged exome sequencing data from the Penn Medicine Biobank (PMBB) to associate carriage of TM6SF2 variants with electronic health records (EHR) in 44,296 unselected participants. Participants with a history of hepatitis B/C infection or alcohol-related disorders were excluded. Liver phenotypes were identified in participants using ICD-10 codes and imaging data, and non-carriers of a TM6SF2 variant were controls. Phenome-wide association studies were replicated in the UK Biobank and statistical analyses were done using R and PRISM.

**Results:** Two non-synonymous variants, rs58542926 (E167K) and rs187429064 (L156P), and one rare stop-gain predicted-loss-of-function (pLOF) variant rs1272943322 (W35X) were significantly associated with physician-diagnosed NAFLD and NASH. Of these, an increased risk of HCC was pronounced in homozygotes of E167K and L156P. A PheWAS confirmed these associations to liver cancer in the UK Biobank. A BMI>30kg/m and carriage of PNPLA3 rs738409:G, attenuated the effects of liver disease, while type 2 diabetes (T2DM) was an insignificant risk factor. E167K and L156P carriers exhibited higher CT-derived hepatic fat scores in an allele-dose manner compared to non-carriers. ALT was increased in E167K heterozygotes, while cholesterol was significantly lower in both heterozygote carriers of E167K and L156P.

**Discussion:** We identified a novel stop-gain pLOF variant in TM6SF2 associated with liver disease not previously reported, while E167K and L156P carriers had a higher risk of liver cancer and hepatic fat accumulation. Obesity was correlated with increased steatosis and HCC, while type 2 diabetes was not a significant risk factor amongst diseased-carriers. Further investigations on liver-related mortality in TM6SF2 carriers is crucial to improve risk-stratification amongst NAFLD patients.

#### P18: Doppler-guided transanal hemorrhoidal dearterialization via mucopexy is effective, safe, and improves quality of life in Canadian patients

##### Andrew Dorovenis^1^, Mihail Kancharla^1^, Anish Engineer^1^, Aneesh Kapoor^1^, Demetra Lalos^1^, Ashwin Maharaj^2^

###### ^1^Royal College of Surgeons in Ireland; ^2^ProctoCAN

####### **Correspondence:** Andrew Dorovenis; Demetra Lalos

*BMC Proceedings 2023*, **17(Suppl 17):**P18

**Aim:** Haemorrhoidal disease is a common condition impacting Canadians. Surgical management of haemorrhoids includes 4 main approaches, all with differing clinical outcomes. This study evaluated post-operative results of the first Canadian patients who underwent Transanal Hemorrhoidal Dearterialization (THD) with mucopexy.

**Method:** This cohort study included patients with grades 3/4 haemorrhoids attending the Thornhill Endoscopy Center (Toronto, Canada) that underwent THD with mucopexy from June 2018 to March 2020. Patients were contacted via phone between 6 months to 2 years post-op. We collected baseline characteristics, previous medical and surgical treatments for haemorrhoids, number of weeks missed from work, pre and postoperative quality of life scores, failure and relapse rate, and haemorrhoid symptom severity scale by Thaha et al (2009). This scale evaluated pruritus, pain, prolapse, bleeding, soiling, and incontinence to gas.

**Results:** In total, 42 patients with a mean age 51.5 years and 21% female were used. Prior to surgery, 85.7% of patients experienced prolapse and 78.6% experienced bleeding. The average Thaha et. al score was 6.4 (out of 19). Half of patients noted that haemorrhoids impacted their quality of life in an unbearable way. Following the operation, 19% of patients experienced surgical failure, 14.3% of patients experienced recurrence, and 80% were able to return to work within four weeks post-op. THD with mucopexy improved quality of life in 78% of cases.

**Conclusion:** Our study demonstrated that the doppler-guided haemorrhoidal artery ligation with mucopexy technique was successful in relieving pain, improving quality of life, and having a quicker return to work.

#### P19: Effect of Chronic Hyperglycemia on Human Induced Pluripotent Stem Cell (hiPSC) Derived Endothelial Cell Function

##### Hannah Berman, Shane Browne

###### Tissue Engineering Research Group (TERG), Dept. of Anatomy and Regenerative Medicine, RCSI, Dublin 2

####### **Correspondence:** Hannah Berman

*BMC Proceedings 2023*, **17(Suppl 17):**P19

Diabetic foot ulcer (DFU) is a major debilitating complication of diabetes, estimated to occur in up to 34% of diabetic patients during their lifetime. Diabetes and hyperglycaemia are associated with dysfunction in all stages of wound healing, particularly the vascularization stage. Vascularization depends on the migration and proliferation of endothelial cells (ECs) lining the blood vessels, making EC behaviour under hyperglycaemic conditions important to study. The aim of this project was to differentiate and isolate ECs from human induced pluripotent stem cells (hiPSCs) and to assess the effect of chronic hyperglycaemia on iPSC derived EC proliferation and migration. ECs were differentiated and purified from hiPSCs using established differentiation protocols and magnetic activated cell sorting based off the expression of the EC surface maker CD31. ECs derived from hiPSCs (iECS) were maintained in 3 conditions: (1) Euglycemia, (2) Chronic hyperglycaemia: 30 mM Glucose media for 7 days, (3) Acute hyperglycaemia: Euglycemia days 0-7, 30 mM Glucose from day 7 onward The effects of hyperglycaemia on cell migration, metabolic activity and cell proliferation were assessed using a scratch assay, AlamarBlue and PicoGreen assays, respectively. After 7 days iECs cultured in hyperglycemic conditions showed a trend of less migration but no significant difference. iECs in chronic hyperglycemia and euglycemia showed a modest but not statistically significant increase in metabolic activity and increased DNA content from day 2 to day 4. iECs in acute hyperglycemia conditions showed increased metabolic activity and decreased DNA content from day 2 to day 4. While the cell migration assay showed a parallel between what is seen in non-healing DFU and what was shown in the experiment, in future experiments cells may require more long-term culture to see a significant effect on the parameters measured.

#### P20: Effects of IL-8 stimulation on ERK phosphorylation in cellular model for Hereditary Hemorrhagic Telangiectasia

##### Qiuwang Zhang^1^, Adam Lang^2^, Michael Kutryk^1^

###### ^1^University of Toronto; ^2^School of Medicine, RCSI University of Medicine and Health Sciences, Dublin, Ireland

####### **Correspondence:** Adam Lang

*BMC Proceedings 2023*, **17(Suppl 17):**P20

Endoglin is a key protein for endothelium regulation and mutations have been shown to lead to hereditary hemorrhagic telangiectasia (HHT). As HHT is characterized by vascular dysplasia and abnormal inflammation responses, we sought to investigate whether introduction of IL-8 would cause abnormal activation of cellular signalling proteins such as ERK and AKT. Using human umbilical vein endothelial cells (HUVEC) as our non-HHT model, and cells transfected with siRNA to knockdown endoglin acting as our HHT model cells, we compared the effects of IL-8 stimulation on protein expression and phosphorylation. Proteins of interest were extracted and separated via western blot before quantification by secondary fluorescent antibodies. We observed a reduction in the phosphorylation of AKT in HHT model cells treated with IL-8 compared to our non-HHT model which was also treated with IL-8. Additionally, the HHT model cells showed higher phosphorylation of ERK compared to the non-HHT model cells when both samples were treated with IL-8 as well as when neither sample was subjected to IL-8. This provides evidence that abnormal activation of ERK and AKT are part of the pathogenesis of HHT in individuals with endoglin mutations which may help us better understand this disease in the future.

#### P21: Effects of Prehabilitation on patients undergoing major abdominal surgeries for Gastrointestinal cancer treatment: A Systematic Review

##### Samher Jassim^1^, Kathryn McKnight^2^, Michael Newell^1^

###### ^1^Department of Surgery, University of Galway, Ireland; ^2^School of Medicine, University of Limerick

####### **Correspondence:** Kathryn McKnight

*BMC Proceedings 2023*, **17(Suppl 17):**P21

**Backgound:** Gastrointestinal cancers are one of the leading causes of cancer-related deaths worldwide, with surgical interventions at the forefront of management. Aim(s): Present up-to-date evidence surrounding the effects of prehabilitation on patients undergoing abdominal surgery for GI cancer.

**Objective(s):** Identify the effects of prehabilitation on postoperative outcomes, length of hospital stay, mortality, ICU admission and readmissions. We also hope to discuss prehabilitation-related compliance.

**Rationale:** Optimising preoperative health is a well-established aspect of surgical care, but little evidence exists illustrating the specific effects that structured prehabilitation may have on GI cancer patients undergoing abdominal surgery.

**Methods:** A systematic search of multiple electronic databases was performed using a search strategy comprising of relevant keywords and controlled vocabulary. Eight studies were selected for inclusion consisting of a total of 6,006 participants.

**Results:** A lower incidence of postoperative complications along with shorter hospital stays was noted in prehabilitation participants, but higher rates of readmission. Compliance with prehabilitation was affected by factors such as the modality of prehabilitation and supervision.

**Discussion:** Evidence supports that prehabilitation has the potential to benefit GI cancer patients undergoing abdominal surgery. However, our systematic review found a vast variation in the significance of these effects.

**Conclusion:** Major abdominal surgery for cancer patients has a significant physical and mental toll. Future studies require congruence regarding participant selection criteria and intervention protocols to allow precise interpretation of the effects of prehabilitation among this patient cohort.

Keywords: ‘preoperative exercise,’ ‘prehabilitation,’ ‘cancer surgery’, ‘abdominal surgery’, ‘gastrointestinal cancer’, and ‘randomised control trial’

#### P22: Endoscopic procedures of the nose and sinuses - Is a histopathological evaluation always necessary?

##### Maya Madhavan^1^, Magdalena Ostrowska^2^, Maciej Wrobel^2^

###### ^1^ENT Scientific Club at the Department of Otolaryngology and Laryngological Oncology, Collegium Medicum, Bydgoszcz, Poland; ^2^Department of Otolaryngology and Laryngological Oncology, Collegium Medicum Bydgoszcz

####### **Correspondence:** Maya Madhavan

*BMC Proceedings 2023*, **17(Suppl 17):**P22

**Objectives:** The aim of the study is to analyse and diagnose histological specimens taken from patients during endoscopic surgery due to changes in the nose and paranasal sinuses and to verify the initial clinical diagnosis against the final histopathology.

**Methods:** Retrospective analysis of medical files, documentation of all endoscopic procedures performed within 12 months was subjected to retrospective analysis. Analysed indications for surgery, preoperative diagnosis, intraoperative and postoperative diagnosis, as well as the result of the subject’s histopathology were analysed. Three groups of patients with preoperative diagnosis were distinguished: A -chronic sinusitis without polyps 52.23 %, B -chronic sinusitis with polyps 42.28 %, C- nasal cavity and / or sinus tumors 5.47 %.

**Results:** In Group A, postoperative diagnosis of chronic sinusitis without polyps in all cases, confirmed the initial diagnosis, in group B, 2.35% of patients were diagnosed with a malignant neoplasm as a final result and a benign lesion in 2.35% of cases; in group C - neoplasm was confirmed in all cases of the primary tumor, while in the group suspected of recurrence, histological confirmation of the primary clinical diagnosis was obtained in all but one patients.

**Conclusion:** Regardless of the clinical conditions and the original diagnosis in all cases of endoscopic surgery, the collected material should be routinely examined by histopathology. While in cases of lesions suspected of a proliferative process, it is an obvious procedure, in the group of patients with a clinical diagnosis of chronic sinusitis with polyps, the presence of coexisting neoplastic lesions should be taken into account.

#### P23: Evaluating patient engagement with community resources after participation in social prescribing during the COVID-19 pandemic: A mixed methods evaluation of the LinkMM randomised control trial

##### Natalie Mack^1^, Bridget Kiely^2^, Susan M Smith^3^

###### ^1^School of Medicine, Royal College of Surgeons in Ireland; ^2^HRB Centre for Primary Care Research, Royal College of Surgeons in Ireland; ^3^Discipline of Public Health and Primary Care, Trinity College, Dublin 2

####### **Correspondence:** Natalie Mack

*BMC Proceedings 2023*, **17(Suppl 17):**P23

**Introduction:** The evidence base for social prescribing in Ireland is building. Although social prescribing aims to get patients involved with community resources, the extent to which patients connect with the resources they are referred to remains largely unknown. This report presents an initial analysis of a wider process evaluation of the LinkMM trial. The objectives of this study were to:1) Summarise the available community resources in the areas in which the LinkMM intervention was implemented 2) Describe the most common resources participants were referred to 3) Assess if people engaged with these resources 4) Describe participant experiences with these community resources.

**Methods:** A total of 240 patients with multimorbidity participated in a randomised control trial of social prescribing link workers based in 13 general practices serving urban, deprived areas of Ireland between July 2020 and January 2021. Quantitative data on available community resources mapped by link workers, patient referrals to resources from a patient database, and patient engagement with resources from a one-year follow-up survey were analysed using descriptive statistics. Semi-structured interviews were conducted with 25 participants six to eight weeks after trial completion. Qualitative interview data on community resource use and experiences underwent thematic analysis with NVivo.

**Results:** Link workers identified a range of available resources, but primarily referred participants to chronic disease-specific supports, mental health services, and social activities. One year post-intervention, participants were mostly engaging with health and fitness services as well as personal hobbies. Thematic analysis revealed that participant barriers to resource engagement included a lack of motivation, limited availability with personal schedules, and COVID-19 pandemic restrictions.

**Discussion:** Patients engaged most with social prescribing recommendations centred around individual self-help. Lower community resource engagement may have been influenced by COVID-19 pandemic restrictions. Future studies should investigate patient engagement with resources over time to determine the long-term impact of social prescribing.

#### P24: Evaluation of a novel outpatient project for Peripheral Arterial Disease (PAD) and diabetic foot disease at Sunnybrook Health Sciences Centre: Vascular Limb Preservation Program (VLPP). A Quality Improvement Study

##### Elena Colussi-Pelaez^1^, Giuseppe Papia^2^

###### ^1^Royal College of Surgeons in Ireland; ^2^University of Toronto

####### **Correspondence:** Elena Colussi-Pelaez

*BMC Proceedings 2023*, **17(Suppl 17):**P24

**Introduction**: As part of a province-wide lower limb preservation program, the vascular surgeons at Sunnybrook Health Sciences Centre in Toronto, Ontario initiated a novel outpatient Vascular Limb Preservation Program (VLPP) to treat wounds and prevent lower limb amputations caused by peripheral arterial disease (PAD) and diabetes mellitus. The purpose of this quality improvement study is to describe the program and determine if it has achieved its primary outcomes of reducing referral times, reducing time to intervention and treatment, and determining proof of concept of this model of care.

**Methods:** A medical record review of all patients who were referred to, and attended, the VLPP from April 1, 2021, until February 10, 2022, was conducted. The patient’s medical records were accessed using SunnyCare, the electronical medical record system at Sunnybrook Health Sciences Centre and recorded. The variables recorded include basic patient information and demographics, referral information, admission data, results from PAD assessment with the nurse practitioner, investigations performed and visit decisions.

**Results:** Data collection is ongoing, a total of 86 patients have been completed. Next set of data collection would include collecting and analysing the VascuQoL-6 questionnaires distributed to each patient at the clinic as well as the satisfaction questionnaire distributed to referring physicians regarding this new paradigm of care in this population of patients. The amputation rates will also be analysed in April 2022, which marks the 1-year point of the project commencement.

**Conclusion:** Early results of this study suggest that the time to referral to specialised vascular care is satisfactory with the current guidelines. The prompt referral time along with the multidisciplinary approach of the VLPP will help in lowering non-traumatic lower limb amputation rates, reducing hospital admissions, and lowering healthcare costs.

#### P25: Exercise and endometriosis: is there a promising future?

##### Kathryn McKnight^1^, Oluwadamilola Omotosho^1^, Amanda Cotter^2^

###### ^1^School of Medicine, University of Limerick; ^2^University Maternity Hospital Limerick (UMHL), Limerick, Ireland

####### **Correspondence:** Kathryn McKnight

*BMC Proceedings 2023*, **17(Suppl 17):**P25

**Background:** Endometriosis is the leading cause of chronic pelvic pain in women of reproductive age with debilitating effects on quality of life, yet no cure exists. Exercise yields the potential in providing women with a non-invasive, non-pharmacological method of symptom control. Scientific literature has alluded to exercise being a favourable factor in the management of endometriosis-related symptoms. Moreover, current clinical guidelines for endometriosis fail to reflect the aforementioned benefits of exercise.

**Aim(s):** Present up-to-date knowledge regarding how exercise may contribute to the management of endometriosis-related symptoms.

**Objective(s):** Discuss: 1) The pathophysiology surrounding exercise and endometriosis. 2) The role of exercise in endometriosis symptom control.

**Methods:** A search strategy using the terms ‘endometriosis’, ‘endometriomas’, ‘exercise’, and ‘physical activity’ was devised. Pubmed, Medline, Cochrane reviews, and Embase were reviewed. Interventional studies, within-subjects studies, randomized-control trials, systematic reviews, meta-analysis, cohort-studies, and publication since 2000 were included. Non-English publications and non-human studies were excluded.

**Results:** Numerous studies have suggested positive effects for endometriosis patients who performed exercise exclusively or in conjunction with other therapies. Improvements in pain levels, quality of life, anxiety, and depression were noted.

**Conclusion:** Achieving symptom control in women with endometriosis is a continuing challenge. Evidence from interventional studies supports exercise as a potentially beneficial modality in management of endometriosis related symptoms as well as a synergy between exercise and hormonal therapies for management. A body of evidence regarding the processes mediating endometriosis exist and were summarised in this review to further support the manner by which exercise can produce its positive effects. The current paucity of high-quality robust studies investigating these aspects of endometriosis management is an apparent obstacle to progression in this area. For clinicians to incorporate exercise in managing endometriosis, clear recommendations regarding advice and benefits are needed.

Keywords: *physical activity; exercise; endometriosis; endometriosis symptom management; preventative medicine; lifestyle medicine*

#### P26: Factors associated with in-hospital mortality in acute pulmonary embolism: a retrospective multicenter cohort study

##### Grzegorz Procyk^1^, Paweł Kurzyna^1^, Karolina Jasińska-Gniadzik^1^, Dominika Rymaszewska^1^, Julia Smyk^1^, Piotr Szwed^1^, Marcin Wasilewski^1^, Rafał Wolański^1^, Aleksandra Gąsecka^1^, Arkadiusz Pietrasik^1^, Marcin Kurzyna^2^

###### ^1^1st Chair and Department of Cardiology, Medical University of Warsaw; ^2^Department of Pulmonary Circulation, Thromboembolic Diseases and Cardiology, Centre of Postgraduate Medical Education, European Health Centre Otwock

####### **Correspondence:** Grzegorz Procyk

*BMC Proceedings 2023*, **17(Suppl 17):**P26

**Introduction:** Pulmonary embolism (PE) is an acute cardiovascular condition associated with high mortality. Conditions related to increased PE mortality are insufficiently characterized. We report the results of a multicenter retrospective study characterizing factors affecting in-hospital mortality in PE patients.

**Methods:** Patients diagnosed with PE between 09.2017-12.2021 in academic centers in Poland were included. Clinical and treatment data were obtained from medical records. Patients’ outcomes were assessed until death or hospital discharge. Mann-Whitney U test was used for nonparametric continuous variables and Fisher’s exact test was performed for categorical variables.

**Results:** The study cohort included 580 patients (49.3% male). The PE risk assessed according to ESC Guidelines was low in 205 (35.3%), intermediate-low in 240 (41.4%), intermediate-high in 103 (17.8%), and high in 32 (5.5%) patients. Women were older than men (median 72.0 years, IQR 62.0-83.0 vs. 65.0 years, IQR 53.0-74.3; p<0.001). Gender had no impact on in-hospital mortality (male/female OR=0.70; 95%CI 0.40-1.21; p=0.212). Patients with active or previous COVID-19 infection presented with lower Pulmonary Embolism Severity Index than COVID-19 negative patients (84.5, IQR 58.25-105.3 vs. 92.0, IQR 71.0-120.0; p=0.015), which might be due to age differences (59.0, IQR 45.8-69.5 vs. 69.0, IQR 57.0-80.0; p<0.001). Nevertheless, COVID-19 status did not affect in-hospital mortality (OR=2.13; 95%CI 0.54-9.19; p=0.413). Neoplastic disease was not associated with increased in-hospital mortality (OR=1.57; 95%CI 0.87-2.92; p=0.180). However, lung cancer increased in-hospital mortality (OR=4.19; 95%CI 1.61-10.94; p=0.009). The following symptoms on admission increased in-hospital mortality: circulatory arrest (OR=109.1; 95%CI 15.71-1212; p<0.001), tachypnoea (OR=5.33; 95% CI 2.86-9.89; p<0.001), oxygen saturation<90% (OR=2.98; 95%CI 1.58-5.62; p<0.001), and syncope (OR=2.39; 95%CI 1.07-5.11; p=0.037).

**Discussion:** Our results are consistent with the current knowledge regarding factors associated with in-hospital mortality in PE patients. Regarding novel findings, we found that lung cancer was associated with increased in-hospital mortality, while COVID-19 infection was not.

#### P27: General Practice in the COVID-19 Era: A Qualitative Interview Study

##### Jennifer McKinlay^1^, Colin Bradley^2^

###### ^1^University College Cork; ^2^University College Cork Department of General Practice

####### **Correspondence:** Jennifer McKinlay

*BMC Proceedings 2023*, **17(Suppl 17):**P27

**Introduction:** The COVID-19 pandemic greatly impacted the delivery of healthcare, particularly in general practice. It is important to ascertain how practices in Ireland have adapted to the pandemic and the consequences that have ensued. This study aimed to elucidate what adaptations were made by general practitioners (GPs) to the operations of their practices as well as the perceived advantages or disadvantages of those changes and their implications for the future of general practice.

**Methods:** Qualitative semi-structured interviews were conducted with GPs who practiced both before and during the COVID-19 pandemic. GPs were purposefully sampled based on gender, location (urban vs. rural), and duration in practice. The sample size was determined using Francis et al.’s “ten plus three” approach to identify data saturation. Interviews were conducted via Zoom, transcribed verbatim and analyzed using a descriptive interpretive qualitative approach.

**Results:** 8 GPs were interviewed. Major changes included the use of telemedicine, electronic prescribing and telephone triage systems. Other changes included increased infection control measures and the elimination of walk-in appointments. Perspectives on the advantages and disadvantages of these changes were mixed. All GPs endorsed electronic prescribing and improved infection control measures. More recently qualified GPs found telemedicine to be efficient while experienced GPs generally reported that it increased their workload. Most GPs intend to continue with their adapted practices in the future. The need for greater locum support for GPs and improved timely access to secondary care was also expressed.

**Discussion:** This study describes how GPs have adapted to the COVID-19 pandemic in Ireland. Some changes in practice have been beneficial for patient care delivery while others have increased the workload and may contribute to physician burnout. These results highlight the need for further work in determining how GPs may be best supported in a post-pandemic world.

#### P28: Glucocorticoid taper success after 1 and 2 years of treatment in the Imperial Takayasu Arteritis Cohort

##### Ritu Alapat^1^, Andrew Porter^2^, Charis Pericleous^2^, Taryn Youngstein^2^, Rob Maughan^2^, Justin Mason^2^

###### ^1^School of Medicine, Royal College of Surgeons in Ireland; ^2^National Heart and Lung Institute and Imperial College London

####### **Correspondence:** Ritu Alapat

*BMC Proceedings 2023*, **17(Suppl 17):**P28

**Introduction:** Takayasu Arteritis (TA) is a granulomatous large vessel vasculitis that is treated with high-dose glucocorticoids (GC) and often a Disease Modifying Anti-Rheumatic Drug (DMARD) to induce remission. Subsequently, GC doses are tapered to reduce side-effects. Although current guidelines recommend GC dose be <10mg/day after 1 year, data describing GC tapering in clinical practice is limited. In this study, GC taper success is described, and associated parameters are explored.

**Methodology:** Data from the Imperial College TA Cohort was reviewed; 158 patients followed for 8.4 [5-13.6] yrs. Patients with sufficient data and typical treatment initiation pattern were analysed; commencing either GC monotherapy or GC + DMARD without prior immunosuppression. Primary outcome: <10mg/day GC dose after 1- and 2-years treatment. Secondary outcome: patients achieving same without intensification (second DMARD or biologic agent). Taper-failure and -success groups were compared for baseline parameters and treatment at 5 years. Comparisons are non-parametric, data median [IQR].

**Results:** Of 129 TA cases requiring treatment, 80 (62%) satisfied analysis criteria. Overall, 46 (57.5%) and 59 (73.7%) patients met the primary outcome at year-1 and -2 respectively. Of these, 43 (93.5%) and 46 (78%) did so without treatment intensification. 94% of year-1 taper-success cases maintained this at year-2. In most cases (90.5%), year-2 taper failure was due to inadequate treatment response. Baseline parameters were similar between groups except ESR which was significantly lower in taper-success cases. As expected, initial treatment with GC monotherapy tended to be lower in the taper-success group. At year 5, taper-failure cases had higher GC doses and biologic usage suggesting a more refractory disease course.

**Discussion:** The >10mg/day GC taper target was achieved in 57.5% of patients, increasing to 73.7% by 2 years. Clinical implementation of this target is practical, and will aid in reducing cumulative GC exposure and identifying patients who require treatment intensification.

#### P29: Glycemic control in Gestational Diabetes Mellitus during COVID-19 pandemic

##### Howard Berger^1^, Alexandra Berezowsky^1^, Negar Bagheri^1^, Elena Colussi-Pelaez^2^

###### ^1^Saint Michael's Hospital, Toronto; ^2^Royal College of Surgeons in Ireland

####### **Correspondence:** Elena Colussi-Pelaez

*BMC Proceedings 2023*, **17(Suppl 17):**P29

**Introduction:** About 7-10% of the pregnancies in northern America are complicated by diabetes and 90% of those cases are represented by Gestational diabetes mellitus (GDM). Both maternal and neonatal adverse outcomes of GDM are well established. Maternal adverse outcomes include prolonged labour, operative delivery, perineal injury, and future overt type-2 diabetes. Neonatal adverse outcomes include excessive fetal growth (large for gestational age – LGA), NICU admission, shoulder dystocia, hypoglycemia, respiratory distress syndrome, and electrolyte disturbances. Many of these adverse outcomes are associated with the adequacy of maternal glycemic control. We aimed to evaluate the adequacy of glycemic control in modified virtual GDM management compared with traditional follow up. A secondary aim is to assess the impact of modified virtual GDM management on select perinatal outcomes.

**Methods:** The primary analysis will compare LGA between two groups using a binary logistic regression model. In a secondary analysis we will focus on study group patients and examine whether % of virtual visits is associated with LGA. All statistical analysis will be performed using SPSS software (version 27) with a level of significance 0.05 (p-values < 0.05 will be reported as statistically significant)**.**

**Results:** A total of 706 women were included in the analysis, 172 (24.3%) in the study group and 534 (75.6%) in the control group. The women in the study group had more perineal lacerations and their newborns had more cases of hypoglycemia and birth injury compared to the control group.

**Discussion:** The results indicate that in-person GDM visits are more advantageous and are associated with less cases of perineal lacerations. It is particularly important for women to have in-person visits to follow-up with clinicians and prevent adverse neonatal outcomes from occurring.

#### P30: Healthcare Worker Pay - Investigating the Causes of Canada’s Healthcare Worker Shortage

##### Lorcan Cooke^1^, Sahil Gupta^2^, Seema Marwaha^3^, Jack Romanelli^4^

###### ^1^RCSI; ^2^Li Ka Shing Knowledge Institute, St. Michael’s Hospital, Toronto, Canada; ^3^Healthy Debate, Saint Michael's Hospital, Toronto; ^4^Healthy Debate, Li Ka Shing Knowledge Institute, Toronto

####### **Correspondence:** Lorcan Cooke

*BMC Proceedings 2023*, **17(Suppl 17):**P30

**Introduction:** Canada is currently suffering from the greatest healthcare worker (HCW) shortage in its history - 152,000 unfilled positions as of August 2022. These factors have resulted in record-breaking wait times in emergency rooms (ER) of 20.7 hours and many ER closures. HCW unions have tried to negotiate for higher pay, but since 2019 Bill 124 has capped pay increases of some HCW to 1% while Canada currently has 6.9% inflation, effectively slashing their real wages. This research aims to answer what role HCW pay and living expenses plays in this HCW shortage crisis.

**Methods:** The pay and living expenses for 10 HCW jobs were researched for the provinces of Ontario, Alberta and Quebec. Sources, such as government, job search and union websites, were averaged to deduce their pay. By using the “Living Wage” - “the hourly wage a worker needs to earn to cover their basic expenses and participate in their community [SM1] ”4 and comparing to pay it was calculated if HCW roles are financially sustainable. Several semi-structured interviews were conducted with subject matter experts that gave insight on the relative importance of pay to the staff shortage.

**Results:** Poor pay likely deters potential HCW from the field and is instrumental in explaining staff leaving or changing jobs. Poor compensation may also demoralize current staff when working alongside colleagues being paid more for similar work as with nursing agencies. Poor pay affects some health professions more than others.

**Discussion:** HCW pay and living expenses play a moderate role in the staff shortage by limiting the number of new HCW, and incentivizing switching to fields with higher compensation. This study will help inform politicians of the factors causing the staff shortage and suggest remediation strategies. Ultimately, this research may help to solve the staff shortage and prevent unnecessary harm.

#### P31: Internet-based cognitive behavioural therapy for psychiatric problems in cancer survivors: a systematic review and meta-analysis of randomised controlled trials

##### Valerie Josephine Dirjayanto^1^, Tazkiya Purwati Ariviani^1^, Nathaniel Gilbert Dyson^2^, Priscilla Geraldine^2^, Muhammad Athallah Arsyaf^1^, Febriyan Satria^1^, Jasmine Virginia Anjani^1^, Raisa Zalfa Meutia Abubakar^1^, Cut Aqilla Rhania^1^

###### ^1^Newcastle University and Universitas Indonesia; ^2^Universitas Indonesia

####### **Correspondence:** Valerie Josephine Dirjayanto

*BMC Proceedings 2023*, **17(Suppl 17):**P31

**Background**: As one of the leading causes of death, cancer contributes to increased prevalence of psychiatric problems in survivors. Cognitive behavioral therapy (CBT) is the current gold standard, but challenges exist due to limited access. Internet-based cognitive behavioral therapy (iCBT) could be more beneficial in overcoming these barriers.

**Aim**: To review the effectiveness of iCBT for psychiatric problems in cancer survivors.

**Methods**: Following PRISMA and registered to PROSPERO, literature search was performed in PubMed, Scopus, CINAHL, and Cochrane, searching for studies implementing iCBT for cancer. Quality of studies were evaluated using the Cochrane Risk of Bias 2.0 tool and converted to AHRQ standards. After qualitative extraction, quantitative analysis of mean differences was performed using Review Manager 5.4 in inverse variance, random-effects model, and whenever appropriate, subgroup and sensitivity analyses were performed.

**Results**: There were 11 studies included with a total of 1,961 participants. Internet-based cognitive behavioral therapy demonstrated promising efficacy in reducing adverse psychosocial conditions in cancer survivors, including anxiety (pooledMD: -1.20[95%CI: -4.60--0.77], p=0.01; I2=59%) and depression (pooledMD: -0.99[95%CI: -1.80--0.19], p=0.02; I2=34%). Lower anxiety inclined towards the subgroup implementing >10 weeks of iCBT (pooledMD: -1.90[95%CI: -4.20-0.40], p=0.11; I2=84%), although shorter intervention yielded more significance (pooledMD: -0.79[95%CI:-1.68-0.10], p=0.08; I2=29%). Similarly, more marked reduction was found towards the >10 weeks iCBT subgroup for depression (pooledMD: -1.95[95%CI:-4.01-0.12], p=0.06; I2=74%). Post-intervention, quality of life also significantly increased (pooled MD: 7.45 [ 95%CI: 1.30-13.60], p=0.02; I2=67%). In addition, indicators of sleep quality, fatigue, physical activity, and healthy dietary habits also improved.

**Conclusion**: Internet-based CBT is a promising solution for improving psychosocial conditions in cancer survivors. We recommend scaling up studies to strengthen the evidence for the future possibility of wide-scope clinical application.

#### P32: Inter-rater reliability of patient and proxy-reports for outcome assessments in stroke: An update of a systematic review

##### Raseel Althawadi^1^, Claire Reimer^1^, Sherlissa Ali-Thompson^2^, Catherine Moran^3^, Anne Hickey^3^

###### ^1^School of Medicine, Royal College of Surgeons in Ireland; ^2^Royal College of Surgeons in Ireland; ^3^Dept. Health Psychology, RCSI University of Medicine & Health Sciences, Dublin, Ireland

####### **Correspondence:** Raseel Althawadi

*BMC Proceedings 2023*, **17(Suppl 17):**P32

**Introduction:** To facilitate the rising global burden and disease-related impairment in stroke, as well as the move towards patient-centered healthcare, PROMs are increasingly used to incorporate the patient perspective and facilitate healthcare decision-making. (1) Many stroke patients with cognitive, motor, or language difficulties are unable to participate in PROMs so surrogate or proxy respondents may respond on their behalf; the reliability of which remains unclear. (2) Therefore, the aim of the present study is to update a 2010 systematic review to investigate the inter-rater reliability of proxy respondents answering PROMs on behalf of stroke patients. (3)

**Methods:** A systematic review of the literature was performed. Studies related to the reliability of proxy respondents in stroke were searched for in CINAHL, Embase, APApsych, and Web of Science between 2008-2022. Duplicate studies were removed using EndNote and data extraction was completed in Covidence. In each study, reliability was assessed using ICCs or k-statistics. The ICC and k-statistic were categorized into poor (≤0 0.40), moderate (0.41-0.60), substantial (0.61-0.80), or excellent (>0.80). Furthermore, the Crowe Critical Appraisal Tool v1.4 (CCAT) was used to appraise the quality of the evidence from the included studies and examine the potential risk of bias.

**Results:** For physical domain measures, the reliability of proxy respondents was moderate to excellent (0.41- >0.8). For measures of cognition, memory and thinking, scores ranged from moderate to substantial (0.40-0.80). The reliability of proxies for measures of communication was poor to substantial and social roles and participation scores were poor to substantial (<0.4-0.80). The reliability of the response of proxies for psychological measures were poor to moderate (<0.40-0.60).

**Discussion:** Proxy respondents are reliable sources for patient-reported outcome measures, however, caution should be used when interpreting more subjective measures like anxiety or depression. Furthermore, the results of the CCATs showed no significant risk of bias.

#### P33: Lock & Key of Recurrent BSEP Deficiency: Quantification and Epitope Identification of anti-BSEP autoantibodies

##### Alyssa Takahashi^1^, Akihiro Asai^2^, Chie Naito^2^, Eriko Kishimoto^2^

###### ^1^Royal College of Surgeons in Ireland; ^2^Cincinnati Children's Hospital Medical Center

####### **Correspondence:** Alyssa Takahashi

*BMC Proceedings 2023*, **17(Suppl 17):**P33

Progressive familial intrahepatic cholestasis 2 (PFIC2) is a hereditary disorder due to mutations in the ABCB11 (BSEP) gene expressed in hepatocytes. BSEP regulates bile acid flow by exporting bile acids into the bile canaliculi; BSEP deficiency can lead to cholestasis and liver failure. The standard treatment for severe liver disease in patients with PFIC2 is a liver transplant; an estimated 8% of transplant recipients develop allo-immunity post-transplant. “Recurrent BSEP deficiency” and graft dysfunction are complications that arise after the transplant recipient develops BSEP autoantibodies in the new liver graft. The cause of these complications is established however the mechanism and detection method for these BSEP autoantibodies remains unknown. Our goal is to establish a reliable diagnostic method to detect anti-BSEP autoantibody presence and quantify it to minimise recurrent BSEP deficiency. As well as investigate the specific epitopes to which these autoantibodies react to. We developed a diagnostic method of ELISA to quantify BSEP autoantibodies present in patient’s serum. This ELISA can be used to monitor titters of antibodies. Portion of BSEP amino acid sequence is coated on 96-well plate. With IRB approval and patient consent, two patients of recurrent BSEP deficiency’s serum were analysed. Both patients had confirmed anti-BSEP autoantibodies in their serum from previously conducted western blotting. Using patient’s serum, we quantified the concentration of autoantibodies to a specific portion (616-746aa) of the BSEP peptide. However, there was inconsistent pattern between the patient’s serum. We found that there was a significant presence of autoantibodies in control serum suggesting these specific autoantibodies do not contribute to clinical manifestations. In conclusion, we developed a quantitative ELISA for detecting serum anti-BSEP autoantibodies that can be used for monitoring post liver transplant patients for “recurrent BSEP deficiency”. However, further experimentation must be conducted to identify the causative epitope(s) of clinical manifestations.

#### P34: Measuring platelet aggregation using differential centrifugal sedimentation

##### Zara Ahmed^1^, Ingmar Schoen^2^

###### ^1^School of Medicine, Royal College of Surgeons in Ireland; ^2^School of Pharmacy and Biomolecular Sciences, RCSI University of Medicine and Health Sciences, Dublin 2

####### **Correspondence:** Zara Ahmed

*BMC Proceedings 2023*, **17(Suppl 17):**P34

The aggregation tendency of platelets is an important indicator for thrombotic risk and is measured by light transmission aggregometry (LTA). However, it does not provide information on aggregate sizes and their distributions. This project focuses on using differential centrifugal sedimentation (DCS) as a novel method to sensitively observe platelet aggregation tendencies. The aim of this project was to determine the feasibility of measuring platelets using a CPS machine and to determine changes in size distribution over time. Platelets were isolated using standard centrifugation technique. Platelets were then either subjected to hyper/hypo-osmotic conditions or they were left to aggregate at intervals of 5 seconds using ADP at 20μM concentration. The disc speed of the CPS machine was set to 8000 rpm. A 2-8% iodixanol gradient was built by the sequential injection of 9x 1.6ml starting with 8% and ending at 2% iodixanol. Samples were injected into the centre of the CPS disk and the machine estimated a particle diameter depending on the sedimentation time. Results showed that the size distribution of resting platelets could be measured by DCS with high reproducibility with a standard error of ≤ 0.01μm. DCS also showed an ability to measure Mean Platelet Volume (MPV) with a weak correlation between MPV calculated by DCS versus a standard haematology cell counter. Finally, DCS revealed that platelet aggregates grew over time by fusing together as aggregates appeared heavier as time left to aggregate increased. In conclusion, our novel method of measuring platelets was very sensitive to the onset of aggregation and has the potential to investigate differences between the action of different platelet agonists and aid future platelet research techniques. We thank the Royal College of Surgeons in Ireland for their support and funding.

#### P35: Mechanical vertebral body augmentation versus conventional augmentation techniques for osteoporotic thoracolumbar compression fractures: a systematic review and meta-analysis of outcomes

##### Matthew Macciacchera^1^, Jake McDonnell^2^

###### ^1^Royal College of Surgeons in Ireland; ^2^The Mater Misericordiae University Hospital

####### **Correspondence:** Matthew Macciacchera

*BMC Proceedings 2023*, **17(Suppl 17):**P35

**Background**: Surgical management of osteoporotic compression fractures (OCF) has traditionally consisted of vertebroplasty or kyphoplasty procedures. Mechanical percutaneous vertebral body augmentation (MVPA) systems have recently been introduced as alternatives to traditional methods. However, the effectiveness of MVPA systems versus conventional augmentation techniques for OCFs remains unclear. This serves as the premise for our study.

**Methods**: A systematic review and meta-analysis was conducted as per the Preferred Reporting Items for Systematic Reviews and Meta-Analyses (PRISMA) guidelines. Studies of interest included randomized controlled trials (RCTs) which directly compared patient outcomes following kyphoplasty or vertebroplasty to patients treated with an MVPA system. Clinical and radiological findings were collated and compared for significance between cohorts.

**Results**: 7 RCTs were identified with 1296 patients total. The mean age was 72.5 years. 639 patients underwent kyphoplasty, 88 underwent vertebroplasty, and 569 underwent mechanical vertebral body augmentation using an MPVA system. MVPAs showed similar efficacy for restoration of vertebral body height (p=0.18), total complications (p=0.27), cement extravasation (p=0.31) and device-related complications (p=0.31). MVPAs also showed reduced rates of all new fractures (16.4% vs 22.2%; p=0.17) and adjacent fractures (13.3% vs 14.9%; p=0.23), with improved visual analogue scale (VAS) scores at 6-months (p=0.13).

**Conclusion**: The results of this meta-analysis depict MPVA systems are an efficient alternative to traditional vertebroplasty and kyphoplasty for the treatment of OCFs . Nevertheless, further robust evidence with long-term follow-up data is needed to establish a true benefit over traditional methods, in addition to long-term cost benefit analyses. Keywords: spine surgery; osteoporosis; vertebral body augmentation; comparative; outcomes

#### P36: Modeling and manufacturing a hydrogel-based regenerative contact lens using patient-specific corneal surface data

##### Mert Egemen Caliskan^1^, Semih Ceylan^1^, Eray Atalay^2^

###### ^1^Eskisehir Osmangazi University Medical School; ^2^Eskisehir Osmangazi University Faculty of Medicine Department of Ophthalmology

####### **Correspondence:** Mert Egemen Caliskan

*BMC Proceedings 2023*, **17(Suppl 17):**P36

**Introduction**: Current topical regenerative treatments such as topical autologous serum or platelet-rich-plasma (PRP) are rapidly cleared from the ocular surface resulting in limited interaction of their constituent growth factors with their respective receptors. Therefore, new approaches are necessary to deliver growth factors more efficiently to the ocular surface. Encapsulation of biological products with hydrogel biomaterials is a promising approach for sustained and controlled delivery of growth factors. The aim of this study is to present a methodology for manufacturing a patient-specific regenerative hydrogel lens using 3D corneal tomography data.

**Materials & Methods**: A volunteer subject underwent Pentacam HR imaging and anterior corneal surface elevation data was exported in a CSV file format. The point cloud was imported into Matlab and quadratic curve-fitting was employed to generate data for missing points up to a 7mm radius. MeshLab was then used for surface reconstruction using the ball pivoting algorithm. Surface data was saved as an STL file and imported into Ansys Design Modeler to design the top and bottom contact lens master molds.

**Results**: Master molds were printed using a 3D SLA Printer and were subsequently used to manufacture the negative PDMS molds. Pre-polymer GelMA 10% solution combined with 10% PRP was poured onto the PDMS mold and photopolymerized under visible light (450 – 550nm) for 8 minutes. Patient-specific contact lenses with varying central thicknesses were successfully manufactured. The manufactured hydrogel contact lenses had good durability and preserved its shape while handling.

**Discussion**: Herein we propose a simple methodology to manufacture PRP-encapsulated hydrogel contact lenses using patient-specific clinical imaging data. Using our methodology, the thickness of the regenerative contact lens can easily be tailored to accommodate for the required time of degradation.

#### P37: Modified clinical and ethical construct for patients with acute alcoholic hepatitis undergoing liver transplantation at Yale

##### Kassandra Gressmann^1^, Ramesh Batra^2^

###### ^1^Royal College of Surgeons in Ireland; ^2^Surgical director, liver transplantation, Yale New England Health, New England, Connecticut, United States of America

####### Correspondence: Kassandra Gressmann

*BMC Proceedings 2023*, **17(Suppl 17):**P37

Background: Alcohol-related liver disease is the leading indication for liver transplantation in the United States. Most transplant centres' transplant listing eligibility criterion require patients meet at least 6 months of alcohol sobriety. However, this can be unrealistic and unfavourable for patients with severe acute alcoholic hepatitis. In October 2020, Yale-New Haven Organ Transplant Center implemented adjusted criteria to evaluate previously ineligible candidates for liver transplantation with goal of enabling patient survival, reducing transplant waiting-list mortality, and improving outcomes. Methods:Patients with alcoholic hepatitis and/or cirrhosis who failed medical therapy and were unlikely to improve without a liver transplantation underwent psychosocial evaluation. Candidacy was considered if they had adequate insight, strong social support, complied with care, and had no prior alcohol-related illness. The main exclusion criteria included poor insight, multiple prior alcohol relapses, active substance use, uncontrolled psychiatric illness, and previous non-compliance. Results:18 patients were evaluated for liver transplant listing using the updated criteria between October 2020 and August 2022, aged 35 to 66 years, and 7 were female. 8 were declined due to psychosocial factors, 3 met eligibility criteria but died before listing, and 6 met criteria and were listed. Of those listed, 4 have received liver transplants, with an average waiting list time of 4.8 days. Prior to listing, patients were sober for 94.8 days. At time of writing, all had functional grafts with no alcohol relapse. Conclusions:Ethical complexity in organ transplant stems from the responsibility of the transplant centre to pair maximal recipient benefit with ethical utilisation to also reflect good organ stewardship on behalf of the community. The updated criteria reflect an attempt to balance the urgency, utility, and justice of organ transplantation. These criteria demonstrate how the outcomes for patients with alcohol-related liver disease can be optimised without ethical compromise to the donated livers.

#### P38: Natural products and their application in nano-based drug delivery and beyond – Pharmacognosy minireview

##### Pedram Pirghasemi

###### School of Pharmacy and Biomolecular Sciences, RCSI University of Medicine and Health Sciences, Dublin 2

####### **Correspondence:** Pedram Pirghasemi

*BMC Proceedings 2023*, **17(Suppl 17):**P38

A lot of medications used today are of plant origin. Aspirin, an NSAID, was derived initially from Salicin which was isolated from Willow Bark and it is by far one of the most successful drugs ever marketed. First-generation statins such as Lovastatin were similarly isolated from the fungal species Aspergillus Tereus, Atropine, which is an anaesthetic medication used before the surgery to inhibit mucus secretion due to its anticholinergic activity was originally isolated from roots of Datura and belong to a special family of plants named Atropa Belladonna, just to name a few. This mini-view looks at the application of natural products in nano-based drug delivery and its potential use in tissue engineering. A wide search of various databases including PubMed, Scopus, and JACS was conducted focusing on keywords such as “use of Natural Products for drug delivery”, and “Nano based drug delivery and natural products.” The findings of this mini-review showed that approximately 50% of the approved New Chemical Entities (NCE) from Jan 81 - Sept 19 were either from natural products derivative, had natural product pharmacophore or it was unaltered natural product, while the rest were either synthetic, vaccines or biological macromolecules. It was also found that these natural products have a variety of applications in medicine and drug delivery including cardiovascular and neuroprotective, cancer, infectious diseases and even tissue engineering due to their biocompatibility characteristics. It is believed that a combination of nanotechnology and natural products would increase the effectiveness and ultimately bioavailability of these natural compounds and potentially prevent or treat different diseases. The scientific development of nanotechnology can revolutionize the development of formulations based on natural products.

#### P39: Patient attitudes towards same-day thyroidectomy during the COVID-19 pandemic

##### Ravneet Dhillon^1^, Albino Chiodo^2^, Bradley Hubbard^2^, Antoine Eskander^3^, Justine Philteos^4^

###### ^1^Royal College of Surgeons in Ireland; ^2^Michael Garron Hospital - Toronto East Health Network; ^3^Department of Otolaryngology-Head and Neck Surgery, Sunnybrook Health Sciences Centre, University of Toronto, Toronto, Ontario, Canada; ^4^Toronto General Hospital

####### **Correspondence:** Ravneet Dhillon

*BMC Proceedings 2023*, **17(Suppl 17):**P39

The COVID-19 pandemic has imposed constraints on many resources and services, including elective surgeries. Due to the pandemic, thyroidectomies, which traditionally are performed as inpatient procedures, have been cancelled and delayed, resulting in increased wait times and psychological distress for patients. Outpatient thyroidectomies may be a feasible and cost-effective alternative to inpatient procedures. Numerous reviews emphasize the potential of outpatient thyroidectomies as a safe and viable alternative in appropriately selected patients and high-volume hospitals. This study aims to determine patient attitudes towards same-day thyroidectomies during the COVID-19 pandemic, and to determine the proportion of thyroidectomy patients eligible for this procedure at Michael Garron Hospital in Toronto, Canada. 108 eligible participants that were selected and recruited through the Otolaryngology Electronic Medical Records were surveyed via phone interviews. Data collected was quantified using a five-point Likert scale and the Friedman test was used for statistical analysis. Analysis indicates that 65% of patient attitudes were favourable towards same-day thyroidectomies, and patients would avail of such an option if eligible. Although many participants fit the criteria for same-day thyroidectomies, 55% of participants were not knowledgeable about the risks of the procedure. Considering the feasibility and economical benefit of same-day thyroidectomies along with the favourable attitudes of the patient population, same-day thyroidectomies should be introduced. Attention to patient education and the use of standardised protocols will be necessary to ensure quality and patient safety.

#### P40: Patients’ attitudes toward deprescribing and treatment burden: a cross-sectional study

##### Sorcha Mooney^1^, Frank Moriarty^2^, Aisling Croke^3^, Karen Cardwell^4^,. Susan M. Smith^5^, Barbara Clyne^3^

###### ^1^RCSI; ^2^School of Pharmacy and Biomolecular Sciences, RCSI University of Medicine and Health Sciences, Dublin 2; ^3^Department of General Practice, RCSI University of Medicine and Health Sciences, Dublin 2; ^4^Health Information and Quality Authority, Dublin, Ireland; ^5^Department of Public Health and Primary Care, Trinity College, Dublin 2

####### **Correspondence:** Sorcha Mooney

*BMC Proceedings 2023*, **17(Suppl 17):**P40

**Background:** Treatment burden can be high in patients with multimorbidity and polypharmacy. Our objective was to investigate how treatment burden as measured using the Multimorbidity Treatment Burden Questionnaire (MTBQ) is associated with patients' attitude towards deprescribing, as measured by the revised Patients’ Attitude Towards Deprescribing (rPATD).

**Design**: Cross-sectional study utilising data from two studies investigating the integration of pharmacists into general practice in Ireland.

**Methods:** The MTBQ and rPATD were distributed to 156 patients aged ≥65 years and taking ≥10 regular medicines. A total MTBQ score was calculated, and scores were also grouped into four categories (no, low, medium, or high burden). An average score was calculated for each of the rPATD factors; medication Burden, Appropriateness, Concern about stopping, and Involvement. We used descriptive statistics to characterise patients. Multiple regression was used to explore potential associations between attitudes towards deprescribing (rPATD) and treatment burden (MTBQ).

**Results:** Of the 156 participants, 57% were female and the mean age was 77.5 years. A higher MTBQ score was significantly associated with higher rPATD scores in two factors (Burden (p<0.001) and Concern about stopping (p=0.003)) and a lower score for the belief in Appropriateness of medication factor (p<0.001) and satisfaction with medication question (p= 0.001)). MTBQ score was not significantly associated with willingness to stop one or more medications (-0.010 95% CI –0.024 to 0.005, p=0.182).

**Conclusion:** While our analysis did not find an association between an individual’s treatment burden and willingness to stop one or more medications, we did find that higher treatment burden scores were related to patients attitudes towards deprescribing across several factors. This study demonstrates that measuring patients’ treatment burden using the MTBQ can be used to further explore patient preparedness to deprescribe.

**Key Words**: cross-sectional studies; general practice; multimorbidity; treatment burden; polypharmacy; deprescribing.

#### P41: Patterns and trends in first-line anti-tuberculosis drug resistance in a major Malaysian Tertiary Teaching Hospital over a 4-year period (2017-2020)

##### Liz Birdie Shi Yun Ong^1^, Gerald Tze Zhen Ser^2^, Bryan Way Wern Lim^3^, Charles Li Qi Gunn^2^ , Harshini Mahendran^2^, Jane Birdie Shi Qi Ong^3^, Martin Tze Wah Kueh^4^, Nadia Atiya^2^

###### ^1^School of Medicine, Trinity College Dublin; ^2^Faculty of Medicine, University of Malaya; ^3^School of Medicine, University of Galway; ^4^Royal College of Surgeons in Ireland & University College Dublin Malaysia Campus

####### **Correspondence:** Liz Birdie Shi Yun Ong

*BMC Proceedings 2023*, **17(Suppl 17):**P41

**Intro**: Drug-resistant tuberculosis is a significant contributor to antimicrobial resistance globally. Despite tuberculosis (TB) being endemic in Malaysia, there is limited published data from Malaysia on anti-TB drug resistance. This study aims to determine the patterns and trends in first-line anti-TB drug resistance in a major Malaysian tertiary teaching hospital.

**Methods**: A retrospective observational study was conducted on all patients who were diagnosed with culture-confirmed tuberculosis at the University of Malaya Medical Centre, Kuala Lumpur, Malaysia, between 1 January 2017–31 December 2020. Patients were identified from the microbiology laboratory database. The medical records of the patients were reviewed, and the following data were collected using a standardised data collection form: demographic data and first-line anti-TB drug resistance patterns.

**Findings**: Over the 4-year study period, a total of 675 non-duplicate Mycobacterium tuberculosis isolates were identified from the clinical specimens of 675 patients, of whom the majority were men (64.3%) and between 18-40 years of age (39.9%). Only 8.3% of the isolates were resistant to at least one of the first-line anti-TB drugs tested. The most common form of first-line anti-TB drug resistance was resistance to streptomycin (4.0%), followed by resistance to isoniazid (3.6%), resistance to ethambutol (2.7%) and resistance to rifampicin (1.5%). Multidrug-resistant TB (MDR-TB) accounted for only 0.9% of the isolates. Between 2017 and 2020, there was an overall increase in the prevalence of resistance to at least one of the first-line anti-TB drugs (7.4% to 12.0%), rifampicin-resistant TB (RR-TB) (1.3% to 3.3%) and isoniazid-resistant TB (Hr-TB) (3.9% to 4.3%). However, there was no increase in the prevalence of MDR-TB (1.3%).

**Conclusion**: The prevalence of MDR-TB in our study cohort remained low and stable over the 4-year study period. However, given the increase in RR-TB and Hr-TB rates, active and continuous surveillance of trends in anti-TB drug resistance is warranted.

#### P42: Physical activity and hypertension

##### Kathryn McKnight^1^, Andrew O'Regan^2^ , Peter Hayes^3^, . Aoife Keating^4^, . Alexandra Ferrara^5^

###### ^1^University of Limerick; ^2^University of Limerick; ^3^University of Limerick; ^4^University of Limerick; ^5^University of Limerick

####### **Correspondence:** Kathryn McKnight

*BMC Proceedings 2023*, **17(Suppl 17):**P42

**Background:** Hypertension and physical inactivity are leading causes of premature mortality. While both are modifiable risk factors for cardiovascular disease, their prevalence remains high. Aim(s): Present up to date knowledge from scientific studies that underpin the role of Physical Activity (PA) in hypertension management.

**Objective(s): Discuss:** The epidemiology of PA and hypertension,the role of PA in hypertension and the pathophysiology surrounding PA and blood pressure. Rationale: Scientific advances have contributed to understanding of how PA improves blood pressure and the clinically relevant ambulatory blood pressure, but this is not reflected in hypertension guidelines for clinical management of hypertension.

**Search strategy:** A search strategy using the terms ‘hypertension’, ‘high blood pressure’, ‘exercise’, ‘physical activity’, ‘aerobic exercise’, ‘isometric exercise’ was devised. Pubmed was reviewed yielding a large quantity of papers. Inclusion criteria: Interventional studies published in last 10 years. Exclusion criteria: Non-English publications, RCT protocols, non-randomised studies.

**Results:** Longitudinal studies demonstrate a protective effect of higher PA levels as well as higher levels of cardiorespiratory fitness. Interventional studies report improvements in blood pressure because of aerobic and resistance training at different doses and intensities; the improvements in some studies were greatest among groups with established hypertensions; the effect was observed for groups with treatment-resistant hypertension also, a clinically important subgroup.

**Discussion:** Current research provides evidence for the synergy between PA and pharmacotherapy for the treatment of hypertension, providing an opportunity for clinicians to promote PA as an adjunctive treatment as well as a preventative strategy. Conclusion: For clinicians to incorporate PA for hypertension prevention and treatment clear recommendations regarding advice and prescription are required.

#### P43: Predicting the frequency of interventional percutaneous balloon angioplasties on arteriovenous fistulas in hemodialysis patients

##### Jordan Loon^1^, Joel Woodley-Cook^2^

###### ^1^Royal College of Surgeons in Ireland; ^2^Scarborough Health Network

####### **Correspondence:** Jordan Loon

*BMC Proceedings 2023*, **17(Suppl 17):**P43

**Introduction**: Stenosed arteriovenous fistulas are a common cause of haemodialysis access failure that require frequent interventions in the form of percutaneous balloon angioplasty. The aim of this study is to assess the frequency at which we intervene on failing fistulas and what factors have an effect on overall patency, including the tip and line placement of central venous catheters.

**Methods**: Patient medical and imaging records were retrospectively reviewed to include those who have undergone balloon angioplasty since 2015. ANOVA and t-tests were performed to determine the significance of various factors on the frequency of interventions on stenosed fistulas.

**Results**: 299 patients were assessed on the time (mean days) between the creation of their fistula and their first balloon angioplasty. Diabetics required intervention before non-diabetics (694.09 days vs 917.08 days respectively; P=0.033). Patients with the catheter tip placed in the inferior vena cava underwent balloon angioplasty the earliest after fistula creation (130.23 days) compared to the superior vena cava, right atrium and cavoatrial junction, while those with the tip in the superior vena cava had fistulas that remained patent the longest (968.80 days; P= 0.007). The most optimal catheter line positioning for patency was the left internal jugular vein and the least optimal was the femoral vein (1132.80 days vs 142.50 days respectively; p=0.007). The frequency of interventions increased as the number of interventions a patient had received increased for the first 3 balloon angioplasties in those applicable (creation- 1st intervention = 798.45 days, 1st-2nd intervention= 556.52 days, 2nd- 3rd intervention= 364.96 days; P=<0.001).

**Discussion**: Factors such as central venous catheter tip and line placement, number of previous interventions, and diabetic status can be used to predict how frequently arteriovenous fistulas will require intervention by balloon angioplasty in haemodialysis patients.

#### P44: Pilot of a mobile web application to audit and improve hospital cleaning and its effect on bacterial surface contamination of the near-patient area in an intensive care unit

##### Sara Charki^1^, Muireann Fallon^2^, Aoife Kearney^3^

###### ^1^Royal College of Surgeons in Ireland; ^2^Department of Clinical Microbiology, Royal College of Surgeons in Ireland, Beaumont Hospital, Dublin 9, Ireland; ^3^Department of Clinical Microbiology, Royal College of Surgeons in Ireland, Beaumont Hospital, Dublin 9, Ireland

####### **Correspondence:** Sara Charki

*BMC Proceedings 2023*, **17(Suppl 17):**P44

**Background**: The hospital environment is known to contribute to the spread of pathogens causing healthcare-associated infections (HAI). It has been shown that in the hospital, near-patient surfaces are an important source of bacterial contamination, contributing to the spread of HAIs. As decontamination of near-patient surfaces is a crucial tool to decrease HAI, it is imperative to monitor and improve hospital cleaning. In this project, it was hypothesized that digital monitoring of daily cleaning could improve cleaning practices and decrease bacterial contamination of near-patient sites.

**Methods**: A four-week sampling project in two Intensive Care Unit (ICU) wards of one Irish tertiary referral hospital was carried out. Total aerobic contamination (TAC) was assessed using Petrifilm (3M, Ireland). Surface contamination with selected Multi-Drug Resistant Organisms (MDRO) contamination (Methicillin-resistant Staphylococcus aureus, Vancomycin-resistant Enterococci, and Carbapenemase-producing Enterobacterales) was evaluated by swab sampling and culture methods. Cleaning monitoring was carried out for two weeks using a handheld tablet computer, through a digital form.

**Results**: Surface samples were taken over a four-week period in ICU A with and without digital monitoring for a total of 250 and 248 samples respectively. Results show that no digital monitoring had a mean of 1.06 CFU/cm2, whereas the use of digital monitoring produced a mean of 0.52 CFU/cm2. The use of digital monitoring intervention resulted in a 50% reduction in surface contamination. Results also showed a 2.81-fold reduction in hygiene failure rate after introducing digital monitoring. Few MDRO were found during this study.

**Discussion**: In a small-scale intervention study on two Irish critical care units, digital cleaning monitoring was shown to decrease TAC and hygiene failure rates. Larger studies over a longer period would be needed to show a greater, statistically significant decrease, and to draw conclusions on the effect of such an intervention on MDRO and HAI.

#### P45: Post-hoc analysis of the pivot trial to determine long-term re-detachment rates of pneumatic retinopexy and pars plana vitrectomy procedures for the management of primary hematogenous retinal detachment

##### Tugche Pehlivan^1^, Isabela Melos^2^, Rajeev Muni^3^, Roxane Hillier^4^

###### ^1^Royal College of Surgeons in Ireland; ^2^Saint Michael's Hospital, Toronto; ^3^Saint Michael's Hospital, Toronto; ^4^Saint Michael's Hospital, Toronto

####### **Correspondence:** Tugche Pehlivan

*BMC Proceedings 2023*, **17(Suppl 17):**P45

**Introduction**: Pars plana vitrectomy (PPV) remains the globally popular surgical treatment of primary rhegmatogenous retinal detachment (RRD). However, the recent Pneumatic Retinopexy versus Vitrectomy for the Management of primary RDD Outcomes Randomized Trial (PIVOT) suggested that if PIVOT recruitment criteria was met, pneumatic retinopexy (PnR) should be considered the first-line treatment as it offered better visual outcomes and less morbidity than PPV. The purpose of our study was to determine the long-term success of both procedures by comparing long-term re-detachment rates.

**Methods**: Post-hoc analysis of the PIVOT study. PIVOT participants were ineligible if re-intervention to re-attach the retina was performed within one year of the initial procedure. Re-detachment was determined by chart review or telephone interview. The latter was the only accepted method for those with <2 years of follow-up (otherwise marked as unreachable and excluded). A two-tailed t-test was used for statistical significance. The mean follow-up duration was also calculated for each group.

**Results**: There were 176 PIVOT participants with 88 in the PPV and PnR arms. There were 4 ineligible PPV participants (re-intervention) versus 8 PnR participants (7 re-intervention and 1 PIVOT dropout). Of the 84 eligible PPV participants, 22 were unreachable leaving 62 (73.81%) analyzed with 37 chart reviews and 25 phone calls (mean age: 69.10+/-7.93 years). Of the 80 eligible PnR participants, 15 were unreachable leaving 65 (81.25%) analyzed with 33 chart reviews and 32 phone calls (mean age: 70.22+/-10.05). Long term re-detachment rate was 0% and 1.54% (1/65) for PPV and PnR groups respectively (p= 0.33). The mean follow-up duration in years was 4.61+/-2.84 versus 4.48+/-2.66 for PPV and PnR groups respectively.

**Discussion**: There was no statistically significant difference in long-term re-detachment rates for PnR vs PPV. Both procedures are durable treatment options for RRD over an extended period, rarely requiring additional intervention for re-detachment.

#### P46: Relationship between the serotonin-bdnf duo and circadian clock genes in the patients with inflammatory bowel disease

##### Szymon Turkiewicz^1^, Marta Ditmer^2^, Agata Binienda^3^, Agata Gabryelska^4^, Ewa Małecka^5^, Jakub Fichna^6^, Marcin Sochal^7^

###### ^1,2,4,7^Department of Sleep Medicine and Metabolic Disorders, Medical University of Lodz, Poland; ^3,6^Department of Biochemistry, Medical University of Lodz, Poland; ^5^Department of Digestive Tract Diseases, Medical University of Lodz, Poland

####### **Correspondence:** Szymon Turkiewicz

*BMC Proceedings 2023*, **17(Suppl 17):**P46

**Introduction**: Inflammatory bowel disease (IBD) is a group of disorders including ulcerative colitis (UC) and Crohn’s disease (CD). Studies investigate the role of the serotoninergic system, BDNF signaling, and the circadian clock in IBD development; however, there are no publications regarding their overlap. This could be important, in the context of comorbid psychiatric disorders in IBD. The study aims to investigate the molecular basis of sleep disturbances in IBD, including the above pathways.

**Methods**: The study included 81 with IBD (divided using clinical conditions exacerbation (EX) and remission (RE) phases; and subtypes of IBD - CD, UC) and 44 healthy controls (HC). The expression of BDNF, circadian clock genes (such as ARNTL, CLOCK, NPAS2, NR1D1), and SERT (serotonin transporter was determined by qRT-PCR, following RNA isolation and cDNA synthesis. BDNF, serotonin, and SERT serum concentration measurements were performed using ELISA. Funded by Polish Ministry of Education and Science (SKN/SP/536070/2022).

**Results**: IBD group in comparison with HC showed a decreased expression of BDNF (p=0.008), CLOCK (p<0.001), NPAS2 (p=0.001), and NR1D1 (p<0.001) and elevated serotonin and SERT serum concentrations (p=0.026 and p=0.001, respectively). A strong negative correlation was found between ARNTL and BDNF expression in IBD, CD, and UC groups (R<-0.6 and p<0.001 for all), which was independent of the disease duration. In IBD, EX, and CD groups positive correlations were found between serotonin concentration and ARNTL expression (p<0.05), and between SERT and CLOCK expression [s1] (p<0.05).

**Conclusion**: Disruption of the circadian clock is an essential part of IBD. Received outcomes emphasize the important role of serotoninergic and BDNF signaling pathways in the interaction with circadian rhythm in the IBD. The influence of serotonin is significant especially in CD patients and in exacerbation of the IBD. Further research on the molecular basis of comorbid sleep disturbances in IBD are needed.

#### P47: Repurposing auranofin for high-grade serous ovarian cancer therapy

##### Estelle Tran^1^, Farah Abdalbari^2^, Alicia Goyeneche^3^, Carlos Telleria^4^

###### ^1^Royal College of Surgeons in Ireland; ^2,3,4^McGill

####### **Correspondence:** Estelle Tran

*BMC Proceedings 2023*, **17(Suppl 17):**P47

**Introduction**: Ovarian cancer is considered as the deadliest of all gynecological malignancies. Amongst its many subtypes, high-grade serous ovarian cancer (HGSOC) remains the most prevalent in the clinical setting. Auranofin (AF) is an anti-rheumatic drug that has been explored for its potentiality as an anti-cancer agent in several types of malignancies including lung and gastric cancers. Its inhibition of the thioredoxin system in tumor cells has risen the possibility of its benefit as a consolidation therapy against HGSOC for chronic use following standard platinum-based chemotherapy. We hypothesize that Auranofin can induce DNA damage in HGSOC by inhibiting the thioredoxin system.

**Methods**: Two different cells lines were tested on, PEO1 (platinum sensitive) and PEO4 (platinum resistant) cells. HGSOC cells were treated with AF, with and without N-acetyl cysteine (NAC), a powerful antioxidant scavenging for reactive oxygen species. Cytometry was performed to evaluate cell viability. If AF induces cell death in HGSOC cells by inhibiting the thioredoxin system, the addition of NAC should reverse AF’s toxicity. To investigate if DNA damage is induced by AF in both cell lines, western blots were prepared and analyzed for the detection of H2AX, a DNA damage biomarker.

**Results**: In samples treated with 4 mM of AF, with and without NAC, the observed difference in cell viabilities was statistically significant (p <0.05). NAC was able to abrogate AF’s toxicity. PEO4 cells expressed a lower amount of H2AX than PEO1 cells treated with AF. AF’s induction of DNA damage in PEO1 cells seems to be dose related.

**Discussion**: Auranofin can instigate DNA damage in HGSOC through inhibition of the antioxidant system thioredoxin. This offers a new perspective of Auranofin's use to treat this lethal disease and the possibility of a combination of cisplatin and Auranofin to potentiate the killing of both platinum sensitive and resistant HGSOC cells.

#### P48: Risk factors for the formation of reproductive potential disorders in teenage girls

##### Iryna Sokolnyk^1^, Dmytro Koliesnik^2^, Snizhana Sokolnyk^3^, Alla Korniakova^4^

###### ^1^Bukovinian State Medical University (BSMU); ^2^Bukovinian State Medical University (BSMU); ^3^Department of Pediatrics and Medical Genetics, Bukovinian State Medical University (BSMU); ^4^Regional Center for Reproductive Health of Youth, Chernivtsi Regional Children's Clinical Hospital

####### **Correspondence:** Iryna Sokolnyk

*BMC Proceedings 2023*, **17(Suppl 17):**P48

**Introduction:** Reproductive health of a girl begins to form during puberty and depends on the combined influence of socio-biological, hereditary, economic and urban factors. Under inadequate conditions, the body's adaptive capabilities decrease and the risk of reproductive potential disorders increases. Aim: To assess the factors influencing the development of the reproductive function of girls and to identify prognostically significant risk factors for a decrease in reproductive potential.

**Methods:** The groups of social, biological, genealogical anamnesis indicators of 86 girls aged 10-17 years (14.3 ± 2.1) were analysed by the conduct of an open prospective randomised "case-control" study, using social (questionnaire), genealogical (study of heredity in three generations of kinship with regard to gynaecological and somatic severity), mathematical - statistical methods (multifactorial analysis to evaluate the impact of adverse factors; to check the significance of the overall measure of communication - non-parametric Pearson test (χ2) and the odds ratio (OR); the reliability of connection at χ2≥3.84 , p<0.05, OR>1.0[95%CI]).

**Results:** Prognostically significant anamnestic risk factors for the disorders formation of the reproductive potential of teenage girls were identified. Conditionally, they can be divided into two groups: modifying (inflammatory processes of the genital organs, OR=4.22[1.09-11.89], p<0.05; sexual "debut" before the age of 15, OR=3.14[1.02-9.29], p<0.05; stress, OR=2.37[1.05-6.91], p<0.05; extragenital pathology, OR=2.83[1.12-10.22], p<0.05; surgical interventions on ovaries, OR=7.02[3.43-14.52], p<0.05; bad habits, OR=3.47[1.17-9.67], p<0.05; disharmonious physical development, OR=3.31[1.33-8.12], p<0.05; unfavourable social history, OR=2.08[1.22-8.41], p<0.05; infections in childhood, OR=2.53[1.18-10.43], p<0.05) and non-modifying (age of menarche under 11 years, OR=2.69[1.01-9.93], p<0.05; burdened genealogical history for reproductive function on the maternal side, OR=3.04[1.21-11.23], p<0.05; burdened antenatal, OR=5.45[2.11-12.54], p<0.05, and postnatal, OR=3.91[1.09-9.88], p<0.05, periods).

**Conclusion:** The obtained results will make it possible to develop individual preventive measures to improve the reproductive potential based on the identification of determining predictors.

#### P49: Role of endobronchial ultrasound in predicting sarcoidosis based on lymph node size: a retrospective cohort study

##### Michael Roman^1^, Biniam Kidane^2^

###### ^1^Royal College of Surgeons in Ireland; ^2^University of Manitoba

####### **Correspondence:** Michael Roman

*BMC Proceedings 2023*, **17(Suppl 17):**P49

**Introduction/Background:** EBUS-TBNA has been shown to have a high diagnostic yield for sarcoidosis in patients presenting with mediastinal and/ or hilar lymphadenopathy. The goal of this study is to determine if the size of the lymph node being sampled with EBUS-TBNA is a reliable predictive factor for yielding a definitive diagnosis of sarcoidosis.

**Methods:** We retrospectively reviewed patients at a tertiary Canadian center who underwent EBUS for suspected sarcoidosis and/ or were diagnosed with Sarcoidosis between 2013-2018. We extracted data on age, sex, radiological findings, lymph node size and station, number of passes, sedation, complications, and results. Next, we separated patients with a confirmed diagnosis of sarcoidosis and excluded patients who had a cytopathological confirmation of malignancy.

**Results:** We identified 75 patients who had a confirmatory diagnosis of sarcoidosis and/ or underwent EBUS-TBNA for suspected sarcoidosis. The average long-axis diameter for the hilar lymph nodes of pathological positive patients who underwent EBUS was 10.8mm and for the mediastinal lymph nodes the mean was 12.1mm. Data analysis has not yet been completed.

**Conclusion:** No conclusions can be made yet regarding the lymph node sizes being a predictive factor in diagnosing patients with sarcoidosis using EBUS-TBNA, until data analysis is accomplished.

#### P50: Severe malnutrition impacts cd101 expression in neutrophils

##### Miriam Basta^1^, Mehak Thind^2^, Amber Farooqui^3^, Robert Bandsma^1,2,3,4^

###### ^1^Research Student in Translational Medicine, Hospital for Sick Children, Toronto, Canada; ^2^Department of Nutritional Sciences University of Toronto; ^3^Translational Medicine Program, Hospital for Sick Children, Toronto, Canada; ^4^Translational Medicine Program, Hospital for Sick Children, Toronto, Canada

####### **Correspondence:** Miriam Basta

*BMC Proceedings 2023*, **17(Suppl 17):**P50

**Introduction:** Globally, severe malnutrition, specifically undernutrition, is directly and/or indirectly related to mortality in ~45% children under five years. Children with severe malnutrition are highly susceptible to life-threatening infections and often present with hyperinflammation and uncontrolled bacterial infections. Innate immune cells, particularly neutrophils, are critical to control bacterial infections. Therefore, it is expected that dysregulations in the functionality of these cells leads to adverse outcomes. However, little research has been done to understand neutrophil biology in the setting of severe malnutrition.

**Methods:** We fed weanling male mice a low-protein diet (LPD) for 13 days to study the effect of malnutrition on neutrophils. Control mice were fed a normal protein diet (NPD). Both groups were challenged with lipopolysaccharide (LPS) injected intraperitoneally to simulate an acute inflammation (n=6/group). Bone marrow neutrophils were isolated. Western blots were done for maturation markers CD101 (Cluster of Differentiation 101) and Lamin-B2 and granular protein LCN-2 (Lipocalin-2). One-way ANOVA was done to compare NPD control, NPD LPS treated, LPD control, and LPD LPS treated groups.

**Results:** There was a significant difference in LCN-2 when comparing NPD and LPD LPS-treated mice (p<0.01) and CD101 was significant when comparing NPD LPS-treated and LPD (p<0.01). However, LaminB-2 was not significant when compared among any group, though downward trends were observed. Our study provides preliminary results, however further studies are required.

**Discussion:** CD101 is a surface glycoprotein on neutrophils that is upregulated in mature neutrophils. LCN-2 is a secondary granular protein that has bacteriostatic properties. Our study demonstrates CD101 and LCN-2 expression are decreased in LPD and LPD LPS treated mice, suggesting a decrease in mature neutrophils in LPD mice. Impairment in neutrophil maturation can explain the increased susceptibility to uncontrolled infections in the setting of malnutrition and eluding the mechanism can prove to be useful developing novel treatments.

